# The Nogo-C2/Nogo Receptor Complex Regulates the Morphogenesis of Zebrafish Lateral Line Primordium through Modulating the Expression of *dkk1b*, a Wnt Signal Inhibitor

**DOI:** 10.1371/journal.pone.0086345

**Published:** 2014-01-21

**Authors:** Hao-Wei Han, Chih-Ming Chou, Cheng-Ying Chu, Chia-Hsiung Cheng, Chung-Hsiang Yang, Chin-Chun Hung, Pung-Pung Hwang, Shyh-Jye Lee, Yung-Feng Liao, Chang-Jen Huang

**Affiliations:** 1 Institute of Biochemical Sciences, National Taiwan University, Taipei, Taiwan; 2 Institute of Biological Chemistry, Academia Sinica, Taipei, Taiwan; 3 Department of Biochemistry, Taipei Medical University, Taipei, Taiwan; 4 Institute of Cellular and Organismic Biology, Academia Sinica, Taipei, Taiwan; 5 Institute of Zoology, National Taiwan University, Taipei, Taiwan; National Institutes of Health/NICHD, United States of America

## Abstract

The fish lateral line (LL) is a mechanosensory system closely related to the hearing system of higher vertebrates, and it is composed of several neuromasts located on the surface of the fish. These neuromasts can detect changes in external water flow, to assist fish in maintaining a stationary position in a stream. In the present study, we identified a novel function of Nogo/Nogo receptor signaling in the formation of zebrafish neuromasts. Nogo signaling in zebrafish, like that in mammals, involves three ligands and four receptors, as well as three co-receptors (TROY, p75, and LINGO-1). We first demonstrated that Nogo-C2, NgRH1a, p75, and TROY are able to form a Nogo-C2 complex, and that disintegration of this complex causes defective neuromast formation in zebrafish. Time-lapse recording of the CldnB::lynEGFP transgenic line revealed that functional obstruction of the Nogo-C2 complex causes disordered morphogenesis, and reduces rosette formation in the posterior LL (PLL) primordium during migration. Consistent with these findings, hair-cell progenitors were lost from the PLL primordium in *p75*, *TROY*, and *Nogo-C2/NgRH1a* morphants. Notably, the expression levels of *pea3*, a downstream marker of Fgf signaling, and *dkk1b*, a Wnt signaling inhibitor, were both decreased in *p75*, *TROY*, and *Nogo-C2/NgRH1a* morphants; moreover, *dkk1b* mRNA injection could rescue the defects in neuromast formation resulting from knockdown of *p75* or *TROY*. We thus suggest that a novel Nogo-C2 complex, consisting of Nogo-C2, NgRH1a, p75, and TROY, regulates Fgf signaling and *dkk1b* expression, thereby ensuring stable organization of the PLL primordium.

## Introduction

The lateral line (LL) of fish is a mechanosensory system closely related to the hearing system of higher vertebrates [1]. The LL is composed of several neuromasts, which are located on the surface of the fish. These neuromasts detect changes in external water flow, thereby enabling fish to maintain a stationary position in a stream, detect prey, and avoid predators. Neuromasts on the head form the anterior LL (ALL) system, while those on the trunk and tail form the posterior LL (PLL) system. Each neuromast contains three distinct types of cells: the neuromast core contains hair cells, which are surrounded and protected by support and mantle cells. Neuromasts develop from a cell cluster called the primordium, which itself is derived from cephalic placodes. ALL and PLL placodes are located anterior and posterior to the otic placode, respectively [2].

During development, the PLL primordium migrates caudally, and deposits several cell clusters; these subsequently undergo self-organization and differentiation into neuromasts. Migration of the PLL primordium is guided by a ligand-receptor system [3], [4], and activation and crosstalk of three signaling pathways, Wnt, Fgf and Notch, are required for lateral line development in zebrafish [5].

Two chemokine receptors, CXCR4b and CXCR7b, are expressed in the leading and trailing zone of the PLL primordium, respectively. Appropriate spatial expression patterns of chemokine receptors drive the collective migration of the PLL primordium; Wnt and Fgf signaling control its migration by regulating the expression of CXCR4b and CXCR7b in the PLL primordium [6]. Wnt signaling is activated in the leading zone of the migrating primordium, and the leading cells proliferate to replenish the cells lost to neuromast deposition. Recently, two reports have demonstrated that Lef1-dependent Wnt signaling is required for the proliferation of leading cells, but not for primordium migration and organization [7], [8]. Activation of Fgf signaling in the trailing zone is controlled by Wnt-dependent expression and secretion of Fgf3/Fgf10a ligands by leading cells. Fgf signaling initiates the mesenchymal to epithelial transition, enabling protoneuromast formation in migrating PLL primordium [9], [10]. In addition, Fgf signaling is required for hair cell specification and Notch signaling activation in the protoneuromast. Notch signaling is required for supporting cell determination, and maintains correct cell arrangement in maturing neuromasts [10], [11].

An intricate regulatory system delineates the activation zone of Wnt and Fgf signaling in the PLL primordium. Wnt signaling drives Sef expression in order to inhibit Fgf signaling in the leading zone of the PLL primordium. In a reciprocal manner, dkk1b, a secreted inhibitor of Wnt signaling, is expressed by Fgf signaling for limiting the activation zone of Wnt signaling in the PLL primordium [6], [9], [12].

To date, many molecules are known to be involved in the development of the zebrafish LL. For example, the *eya1* gene is essential for hair-cell survival and differentiation, while *tmie* is involved in the maturation, function, and maintenance of hair cells [13], [14]. Furthermore, *lbx2* is required for the migration of the PLL primordium through regulating the expression of *sdf1a* in the horizontal myoseptum [15], and restricted expression of *atoh1a* in the central cell of epithelial rosette is prerequisite for morphogenesis of PLL primordium [11]. Morpholino knockdown of the gene encoding caveolin (Cav)-1 inhibits neuromast maturation in the zebrafish PLL [16].

In mammals, Nogo signaling involves ten molecules: four receptors, three co-receptors, and three ligands. Nogo-A is the best known ligand, and it plays a key role in the inhibition of axonal regrowth after spinal cord injury [17]. It binds to a GPI anchor, the Nogo receptor (NgR), in neurons of the central nervous system (CNS), forming a Nogo complex through association with the three co-receptors: p75, LINGO-1, and TROY [18]–[21]. The zebrafish genome does not encode a homologue of Nogo-A; however, three Rtn4/Nogo-related ligands and four GPI anchor Nogo receptors have been identified in this organism [22]–[25]. In the present study, we cloned three Nogo co-receptors from zebrafish, and characterized their expression patterns during development. Thus, zebrafish is the first non-mammalian vertebrate species reported to possess ten molecules involved in Nogo signaling.

The roles of Nogo-Nogo receptor signaling in zebrafish development have seldom been the subject of study. However, it was recently established that Nogo-B and its receptor, NgBR, affect intersomitic vessel sprouting by modulating the phosphorylation of Akt [26]. Furthermore, neurite outgrowth of the peripheral nervous system is guided by Nogo-NgR signaling in zebrafish. Morpholino knockdown of Rtn4-n/Nogo-γ/Nogo-C1 and NgR resulted in severe defects of neurite outgrowth in the zebrafish PLL [27]. However, it remains unknown whether Nogo-Nogo receptor signaling also participates in the development of the zebrafish LL neuromasts. In the present study, we confirm that knockdown of *p75*, *TRO* *Y*, or *Nogo-C2/NgRH1a* results in a reduction of neuromasts and disorganization of the PLL primordium. Importantly, *p75*, *TRO* *Y* and *Nogo-C2/NgRH1a* morphants exhibited down-regulated expression of *pea3* and *dkk1b* in the PLL primordium; moreover, delivery of *dkk1b* mRNA rescued the neuromast formation defect in *p75* and *TRO* *Y* morphant embryos, suggesting that Nogo signaling coupled with Fgf signaling affects downstream targets during the development of the zebrafish LL system.

## Results

### Isolation of three Nogo co-receptors from zebrafish

To demonstrate the functional role of zebrafish Nogo/Nogo receptor signaling in lateral line development, the genes encoding the three Nogo co-receptors (TROY, p75, and LINGO-1) were first isolated from a zebrafish cDNA library. To identify the zebrafish *p75* gene, we used tBLAST to search GenBank for expression sequence tags (ESTs) related to the coding region of human *p75* (accession no. NM_002507). Full-length zebrafish *p75* cDNA was cloned from the identified EST clones, and was deposited in GenBank with the accession no. GQ983383. Zebrafish *p75* cDNA is predicted to encode a protein of 431 amino acids with 45% sequence identity (61% similarity) to both human and mouse p75 (Fig. S1A). Four cysteine-rich domains (CRD1-4) were identified in the extracellular region, and a death domain at the C-terminal tail of the intracellular region of zebrafish p75.

A similar strategy was used to identify zebrafish *lingo-1* and *troy* cDNAs. Full-length cDNAs of zebrafish *lingo-1* and *troy* were assembled and deposited in GenBank with the accession numbers GQ983382 and EF426728, respectively. Zebrafish *lingo-1* cDNA is predicted to encode a protein of 622 amino acids. As shown in Fig. S1B, the overall amino acid sequence of zebrafish LINGO-1 exhibits 72% identity (86% similarity) with human LINGO-1 and 71% identity (85% similarity) with mouse LINGO-1. Zebrafish *TROY* cDNA is predicted to encode a protein of 356 amino acid residues. The amino acid sequence of zebrafish TROY exhibits 42% identity with mouse and 43% identity with human TROY (56% similarity with both sequences) (Fig. S1C). Like p75, zebrafish TROY also has three CRDs in its extracellular region.

### Expression profiles of Nogo co-receptors at different developmental stages, as revealed by whole-mount *in situ* hybridization

**Figure 1:**
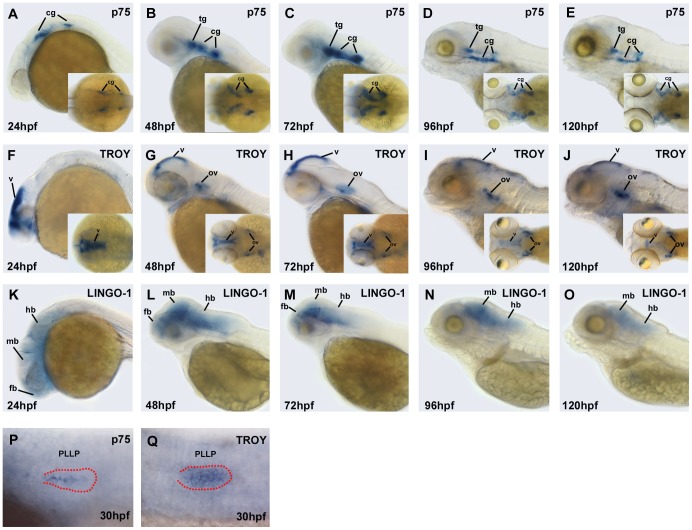
Expression patterns of Nogo co-receptor mRNAs in zebrafish embryos. Whole-mount *in situ* hybridization was performed with antisense probes against *p75* (A–E; P), *TROY* (F–J; Q), and *LINGO-1* (K–O) at the indicated developmental stages. Images were taken from the lateral view with the anterior to the left and the dorsal to the top, or from the dorsal view (inset). The PLL primordium is visible in the embryos with DIC optics, and labeled with red dots in panels (P) and (Q). fb, forebrain; mb, midbrain; hb, hindbrain; tg, trigeminal ganglion; cg, cranial ganglion; v, ventricle; ov, otic vesicle; PLLP, posterior lateral line primordium.

To investigate the temporal and spatial expression patterns of Nogo co-receptors in zebrafish embryos, we synthesized DIG-labeled antisense RNA probes for use in whole-mount *in situ* hybridization. The mRNA expression patterns of the three Nogo co-receptors in zebrafish embryos are shown in Figure 1. At 24∼120 h post-fertilization (hpf), zebrafish *p75* messenger (m)RNA was expressed in trigeminal and cranial ganglia (Fig. 1A–E). Consistent with a previous report [27], we also found that *p75* was primarily expressed in neuronal cells in zebrafish. In 24-hpf embryos, zebrafish *TROY* mRNA was highly expressed in the midbrain ventricle (Fig. 1F). After 24 hpf, *TROY* mRNA was expressed at the edge of the midbrain ventricle and otic vesicle (Fig. 1G–J). On the other hand, zebrafish *LINGO-1* mRNA was expressed in the whole brain in 24-, 48-, and 72-hpf embryos (Fig. 1K–M). After 72 hpf, *LINGO-1* mRNA was predominantly expressed in the midbrain and hindbrain (Fig. 1N–O). The expression pattern of zebrafish *LINGO-1* mRNA during development was similar to that of mammalian *LINGO-1*, which is only expressed in the CNS [18]. Due to the PLL primordium is visible under DIC microscope [28], we also observed that the mRNAs of both *p75* and *TROY* were expressed in certain cells of the migrating PLL primordium at 30 hpf (Fig. 1P–Q), suggesting that Nogo/Nogo receptor signaling may be involved in PLL development in zebrafish.

### Morpholino knockdown of the Nogo co-receptors, p75 and TROY, resulted in significant loss of neuromasts in the zebrafish LL

**Figure 2:**
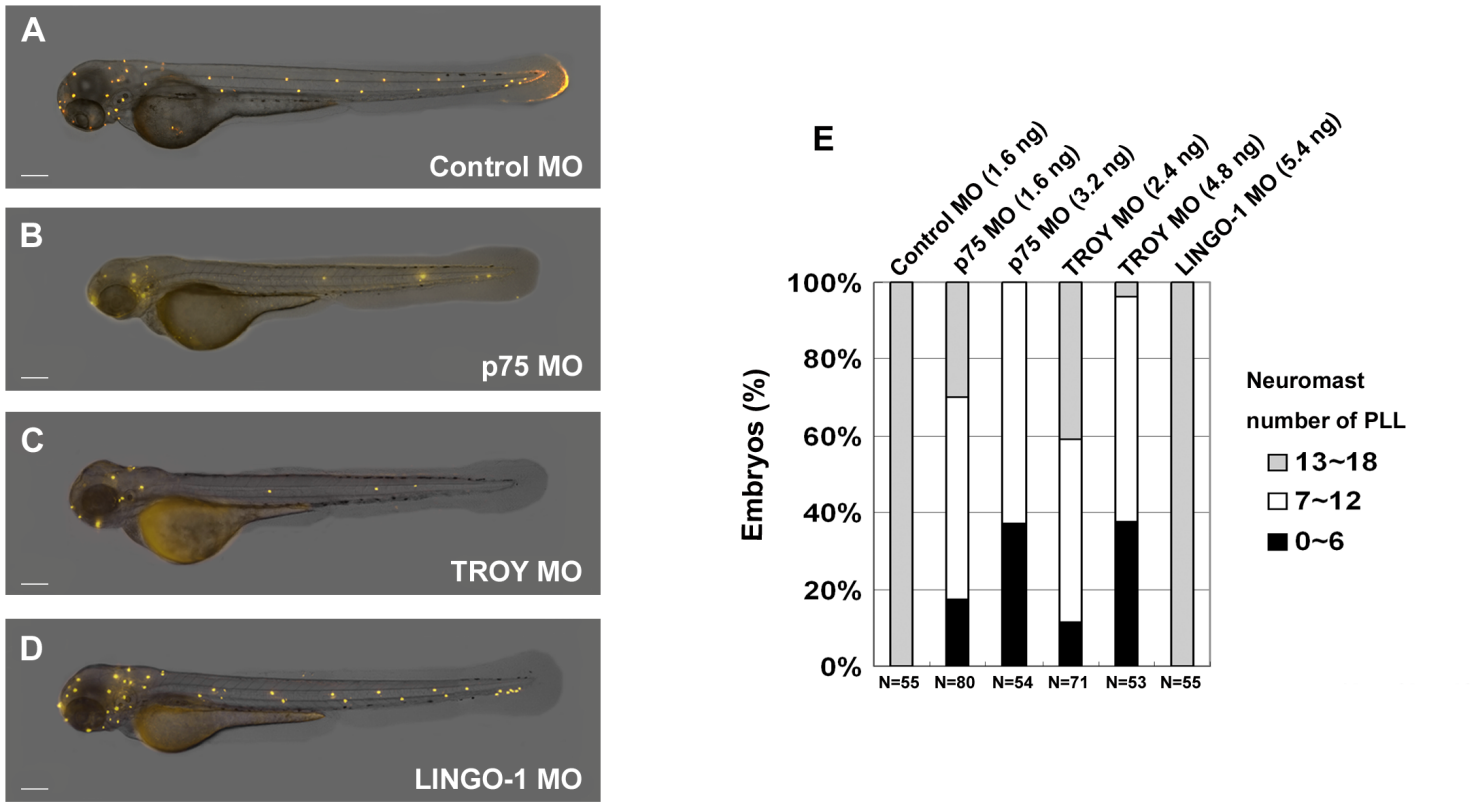
Morpholino knockdown of the Nogo co-receptors *p75* and *TROY* resulted in defects in neuromast formation in the zebrafish lateral line. Zebrafish embryos at the 2∼4-cell stage were injected with morpholinos (MOs) against zebrafish *p75* (B), *TROY* (C), or *LINGO-1* (D). Neuromasts in these morphants were stained with 4-Di-2-ASP at 72 h post-fertilization (hpf). An embryo injected with control MO is shown in panel (A). Images were taken from the lateral view, and the yellow dots indicate neuromasts of the lateral line. Scale bar, 100 µm. A summary of PLL neuromast numbers in MO-injected embryos is shown in panel (E). Sample size (N) and MO dosage used are indicated.

Given that both *p75* and *TROY* are expressed in the migrating primordium (Fig. 1P–Q), we hypothesized that these Nogo co-receptors may play a role in LL development. We injected zebrafish embryos with morpholino oligonucleotides (MO) against a Nogo co-receptor, and then counted the numbers of neuromasts in the ALL and PLL systems of zebrafish at 72 hpf. Live embryos were stained with 4-Di-2-ASP to visualize neuromasts [29]. The PLL system of control embryos or *LINGO-1* morphants contained 15∼18 neuromasts on the trunk at 72 hpf (Fig. 2A, D). However, neuromast numbers in the PLL system were significantly reduced in *p75* and *TROY* morphants (Fig. 2B, C). The percentages of MO-injected embryos with decreased neuromast numbers in the PLL are summarized in Fig. 2E. About 60∼70% of embryos injected with lower doses of *p75*- or *TROY*-MO exhibited an obvious decrease in neuromast numbers at 72 hpf; furthermore, the MOs caused a loss of neuromasts in a dose-dependent manner (Fig. 2E). We also examined neuromast formation in the head region. There were 18∼20 neuromasts on each side of the head in control and *LINGO-1*-MO-injected embryos at 72 hpf (Fig. 2A, D). However, only a few neuromasts formed on each side of the head in *p75* and *TROY* morphants (Fig. 2B, C), suggesting that *p75* and *TROY* knockdown affected the formation of neuromasts in both the ALL and PLL.

To determine the specificities of the MOs used, we created several pCMV-GFP reporter plasmids. The 25-bp target sequence of each morpholino was cloned upstream of the GFP open reading frame (ORF) in the pCMV-GFP reporter plasmid (*mo*-GFP, Fig. S2A–C). As a control, a target sequence bearing 5 mismatches was also cloned into the pCMV-GFP reporter plasmid (MM *mo*-GFP). We co-injected zebrafish embryos with a morpholino and a pCMV-GFP reporter plasmid containing a perfect or mismatched target sequence corresponding to that morpholino (Fig. S2A′–C′ and S2A″–C″). The GFP signal intensity in Fig. S2A′–C′ was decreased as compared to Fig. S2A–C, thereby confirming the specificity of each MO against Nogo co-receptors.

We also used Western blot to confirm that the levels of p75 and TROY protein in zebrafish embryos were reduced in morphants (Fig. S3A–B). As a further control, we used splice-blocking MOs (SB-MOs) to confirm the requirement for p75 and TROY in PLL development in zebrafish (Fig. S3C–D).

### Knockdown of Rtn4-m/Nogo-C2 and Nogo receptor, NgRH1a, caused defects in zebrafish neuromast formation

**Figure 3:**
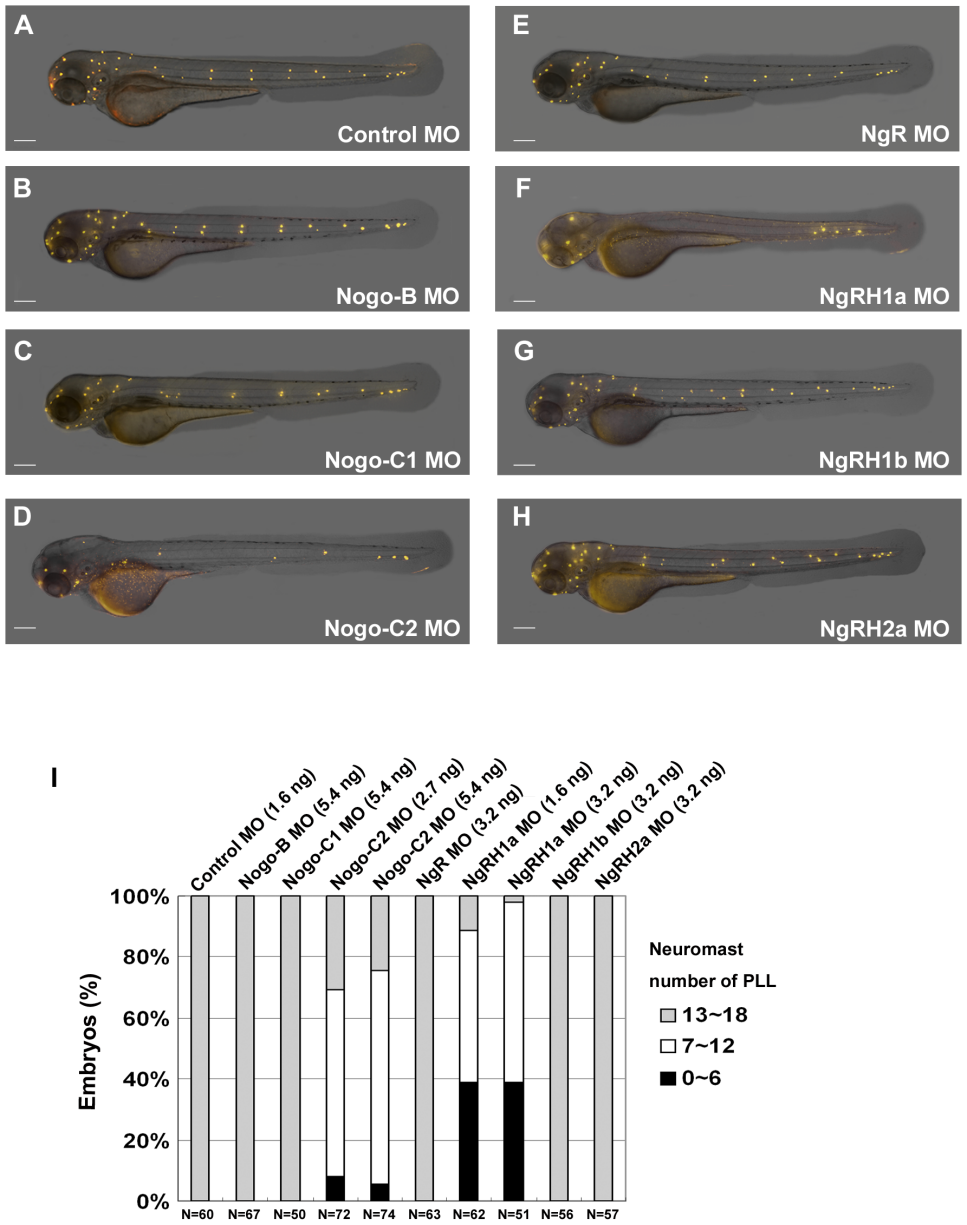
Defects in neuromast formation were observed in both *Rtn4-m/Nogo-C2* and *NgRH1a* morphants. Zebrafish embryos were injected with morpholinos against the indicated zebrafish Nogo-related ligand or Nogo receptor at the 2∼4-cell stage. Neuromast formation in these morphants was observed by staining with 4-Di-2-ASP at 72 h post-fertilization (hpf). A control embryo is shown in panel (A). Images were taken from the lateral view, and the yellow dots indicate neuromasts of the zebrafish lateral line. Scale bar, 100 µm. A summary of PLL neuromast numbers in MO-injected embryo is shown in panel (I). Sample size (N) and MO dosage used are indicated.

We have confirmed that two Nogo co-receptors, p75 and TROY, are required for neuromast formation (Fig. 2). To further elucidate the role of Rtn4/Nogo signaling in LL development, we also examined the effect of injecting embryos with MOs against one of the three Rtn4/Nogo proteins [23], [24] or four Nogo receptors [22]. Injection of embryos with Rtn4-m/Nogo-C2-MO significantly decreased the number of neuromasts at 72 hpf (Fig. 3D), whereas injection with Rtn4-l/Nogo-B-MO (Fig. 3B) or Rtn4-n/Nogo-C1-MO (Fig. 3C) had no effect upon neuromast formation. Similarly, neuromast number was decreased by injection of embryos with NgRH1a-MO (Fig. 3F), but not NgR-MO (Fig. 3E), NgRH1b-MO (Fig. 3G), or NgRH2a-MO (Fig. 3H). The numbers of PLL neuromasts in each morphant are summarized in Fig. 3I. Finally, we confirmed the specificities of each MO against three Rtn4/Nogo proteins and four Nogo receptors (Fig. S4). In addition, we also verified the roles of Nogo-C2 and NgRH1a in neuromast formation by using splice-blocking MOs (Fig. S5).

In summary, we proven that four molecules involved in Nogo signaling, including Nogo-C2, NgRH1a, p75, and TROY, are required for PLL development in zebrafish.

### Association of TROY with p75

We have thus far demonstrated that *p75* and *TROY* are expressed in migrating primordium, and knockdown of either gene results in similar defects in neuromast formation. Furthermore, it has been reported that TROY genetically interacts with DR6, a member of the TNF receptor superfamily, and both are required for CNS angiogenesis and barriergenesis in zebrafish [30]. We thus hypothesized that TROY may interact with p75 in the PLL primordium during caudal migration.

**Figure 4:**
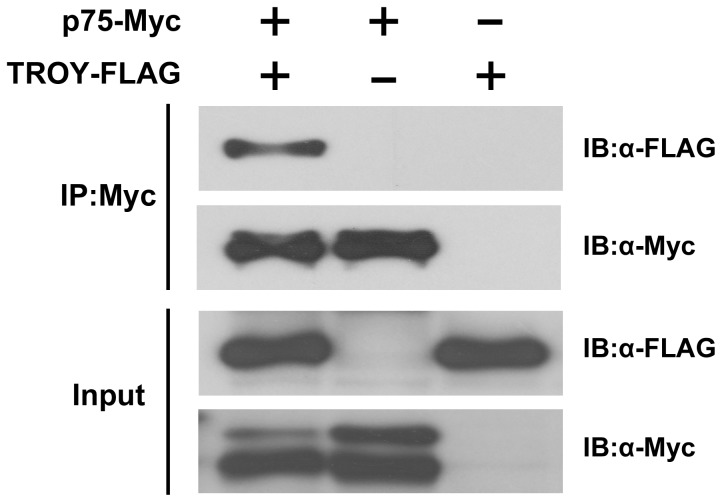
Zebrafish p75 and TROY interact in COS-1 cells. Co-immunoprecipitation was performed using the lysates of COS-1 cells transfected with expression vectors for p75-Myc and/or TROY-FLAG, as indicated. Input was 2% of total protein lysate used in co-immunoprecipitation. The precipitates were subjected to SDS-PAGE and analyzed by immunoblotting with specific antibodies. IP, immunoprecipitation. IB, immunoblot.

To test this hypothesis, we investigated whether p75 and TROY associate *in vitro* by performing a co-immunoprecipitation assay. Two constructs, pcDNA3-p75-Myc and pcDNA3-TROY-FLAG, were co-transfected into COS-1 cells. Cell lysates were prepared and subjected to co-immunoprecipitation using anti-Myc monoclonal antibodies at 48 hours post-transfection. As shown in Figure 4, a TROY-immunoreactive band was strongly detected in Western blots by probing with anti-FLAG antibodies, indicating that p75 and TROY interact with each other in COS-1 cells. It is thus possible that p75 and TROY form a complex in the migrating primordium of zebrafish.

### Zebrafish Rtn4-l/Nogo-B binds to NgR, while Rtn4-m/Nogo-C2 binds to the NgRH1a and NgRH1b receptors

Human Nogo-A has been reported to bind to the NgR, and then recruit other co-receptors to form a Nogo receptor complex in neuronal cells [17]. It is thus critical to determine how binding between the various Nogo proteins and receptors affects neuromast formation in zebrafish. To identify the receptor for zebrafish Nogo proteins, we generated fusion proteins of placenta alkaline phosphatase (AP) and a unique N-terminal region of one zebrafish Rtn4/Nogo-related protein. These fusion proteins were designated as AP-N(1–132)zNogo-B, AP-N(1–25)zNogo-C1, AP-N(1–25)zNogo-C2, and AP-N(11–35)zNogo-C2. The unique N-terminal regions of Rtn4-n/Nogo-C1 and Rtn4-m/Nogo-C2 consisted of 9 and 25 amino acid residues, respectively. To minimize the effect of length on the ligand-receptor binding assay, we generated an AP fusion protein containing 25 amino acid residues of the N-terminus of Rtn4-n/Nogo-C1 (AP-N(1–25)zNogo-C1), and a truncated variant of Rtn4-m/Nogo-C2 (AP-N(11–35)zNogo-C2).

**Figure 5:**
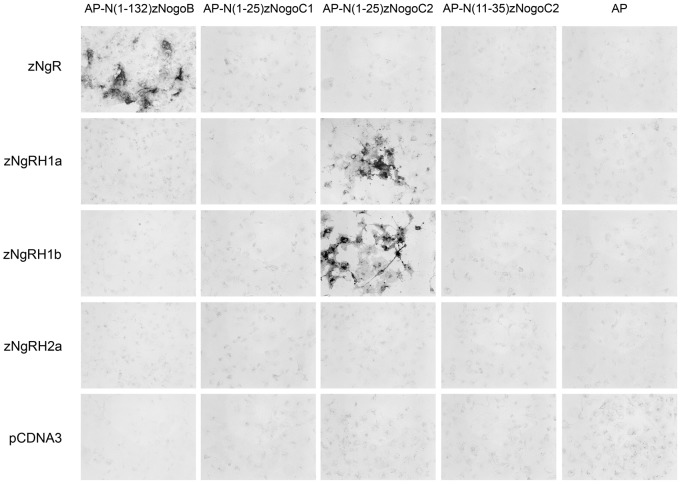
Binding of alkaline phosphatase (AP)-fusion proteins containing N-terminal fragments of Nogo-related proteins to COS-1 cells expressing Nogo receptors. Binding of AP, AP-N(1–132)NogoB, AP-(N1–25)NogoC1, AP-(N1–25)NogoC2, and AP-(N11–35)NogoC2 fusion proteins to COS-1 cells expressing one of the four zebrafish Nogo receptors, or vector alone. AP-(N1–25)NogoC2 binds to both zNgRH1a and zNgRH1b, while AP-N(1–132)NogoB binds to zNgR alone.

For the binding assays, COS-1 cells transfected with pcDNA3 alone or with each of zebrafish Nogo receptor were incubated in conditioned media containing individual AP fusion proteins. After fixation and heat inactivation of endogenous AP, bound AP was detected by the addition of its substrate. As shown in Figure 5, AP-N(1–25)zNogo-C2 bound to zNgRH1a and zNgRH1b, while AP-N(1–132)zNogo-B bound to zNgR. Interestingly, AP-N(11–35)zNogo-C2 did not bind to zNgRH1a or zNgRH1b, suggesting that the first 25 amino acids of the N-terminus of Rtn4-m/Nogo-C2 are required for ligand-receptor binding.

A classical Nogo receptor complex is composed of a ligand, a receptor, and two co-receptors. Based on our MO knockdown, co-immunoprecipitation, and AP ligand-receptor binding assay results, we propose that a novel Rtn4-m/Nogo-C2 complex, consisting of Rtn4-m/Nogo-C2, NgRH1a, TROY, and p75, governs the formation of zebrafish neuromasts.

### Cell proliferation in the PLL primordium is not mediated by Nogo/Nogo receptor signaling during zebrafish LL development

**Figure 6:**
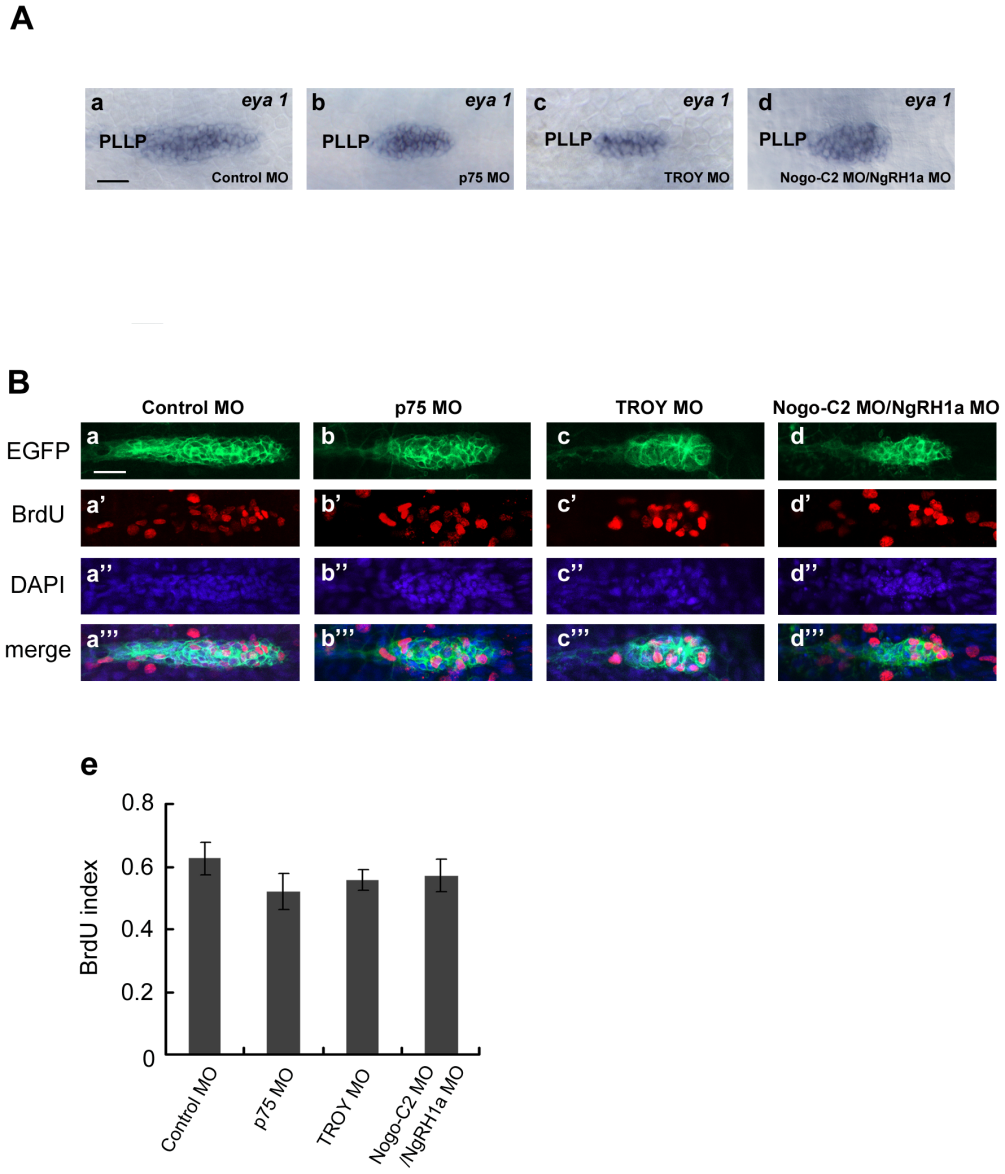
Although reduced in size, the PLL primordium of *p75*, *TROY* and *Nogo-C2/NgRH1a* morphants do not exhibit defects in cell proliferation. (A) PLL primordium in control (panel a) and morphant embryos (panels b–d) were labeled by whole-mount *in situ* hybridization with a probe against *eya1*. PLLP, posterior lateral line primordium. (B) Proliferating cells in PLL primordium were examined by BrdU incorporation. Each MO was injected into the CldnB::lynEGFP transgenic line, and BrdU incorporation assays were subsequently performed from 32.5 hpf. After incorporation, the embryos were fixed and immunostained with anti-GFP and anti-BrdU antibodies. Proliferating cells in the PLL primordium are shown in control embryos (panels a–a′″), and *p75* (b–b′″), *TROY* (c–c′″), and *Nogo-C2*/*NgRH1a* (d–d′″) morphants. Nuclei were also stained with DAPI. Scale bar, 20 µm. The BrdU index (the ratio of BrdU-positive cells/total cells) was determined in MO-injected embryos (panel e).

We proceeded to investigate the mechanisms by which Nogo/Nogo receptor signaling regulate zebrafish LL development. To ensure there are enough cells for neuromast deposition, the cells in the leading zone of the PLL primordium proliferate rhythmically during migration [31]. As shown in Figures 2 and 3, PLL neuromast distribution in *p75*, *TROY*, *Nogo-C2*, and *NgRH1a* morphants was highly similar to that in embryos treated with inhibitors of cell division [8]. In addition, double knockdown of zebrafish *hmx2* and *hmx3*, which are required for cell proliferation in the PLL primordium, resulted in a smaller primordium and consequential neuromast loss [32]. We therefore addressed whether the neuromast loss observed in *p75*, *TROY*, *Nogo-C2*, and *NgRH1a* morphants arose from defective proliferation of cells in the PLL primordium. We first examined the size of the migrating primordium by whole-mount *in situ* hybridization with a probe against *eya1*. As shown in Figure 6A, the migrating primordium was dramatically reduced in size at 30 hpf in *p75* and *TROY* morphants as compared to control embryos, suggesting that cell proliferation may be disrupted in these morphants. Simultaneous knockdown of *Nogo-C2* and *NgRH1a* also reduced the size of the PLL primordium (Fig. 6A, panel d). To assess the levels of proliferation in *p75*, *TROY*, and *Nogo-C2/NgRH1a* morphants, we performed BrdU incorporation assay to label cells in S phase. By employing the transgenic line CldnB::lynEGFP [33], we can observe the proliferating cells in PLL primordium with reduced size during migration. Surprisingly, despite the primordium being almost two-fold smaller than that of control embryos (Fig. 6B, panels a–d), the relative proliferation level of cells in morphant primordium was not significantly different to that of the control (Fig. 6B, panel e); this indicates that neuromast loss in *p75*, *TROY*, *Nogo-C2*, and *NgRH1a* morphants is not due to direct disruption of cell proliferation during PLL primordium migration. On the other hand, to exclude the possibility that the reduction of primordium size was resulted from non-specific MO-induced cell death in the morphants, we performed the TUNEL assays and demonstrated that the number of apoptotic cells was not obviously increased in PLL primordium (Fig. S6).

Previously, we have demonstrated that mRNAs of p75 and TROY were both expressed in PLL primordium (Fig. 1P–Q). Here, we performed whole-mount *in situ* hybridization co-staining with GFP antibody in the CldnB::lynEGFP transgenic line and confirmed that p75, TROY and NgRH1a were expressed in PLL primordium during caudal migration (Fig. S7). In zebrafish, the PLL primordium migrates caudally along horizontal myoseptum during development. In the previous study, we have shown that the EGFP expression driven by Nogo-C2 promoter was localized to skeletal muscles of zebrafish embryos [25], indicating that the endogenous Nogo-C2 is expressed in skeletal muscles in zebrafish. Thus, Nogo-C2 has potential to interact with Nogo receptor complex, consisting of NgRH1a, p75, and TROY, expressed on migrating PLL primordium.

### The Rtn4-m/Nogo-C2 complex regulates primordium organization and rosette formation during PLL development

**Figure 7:**
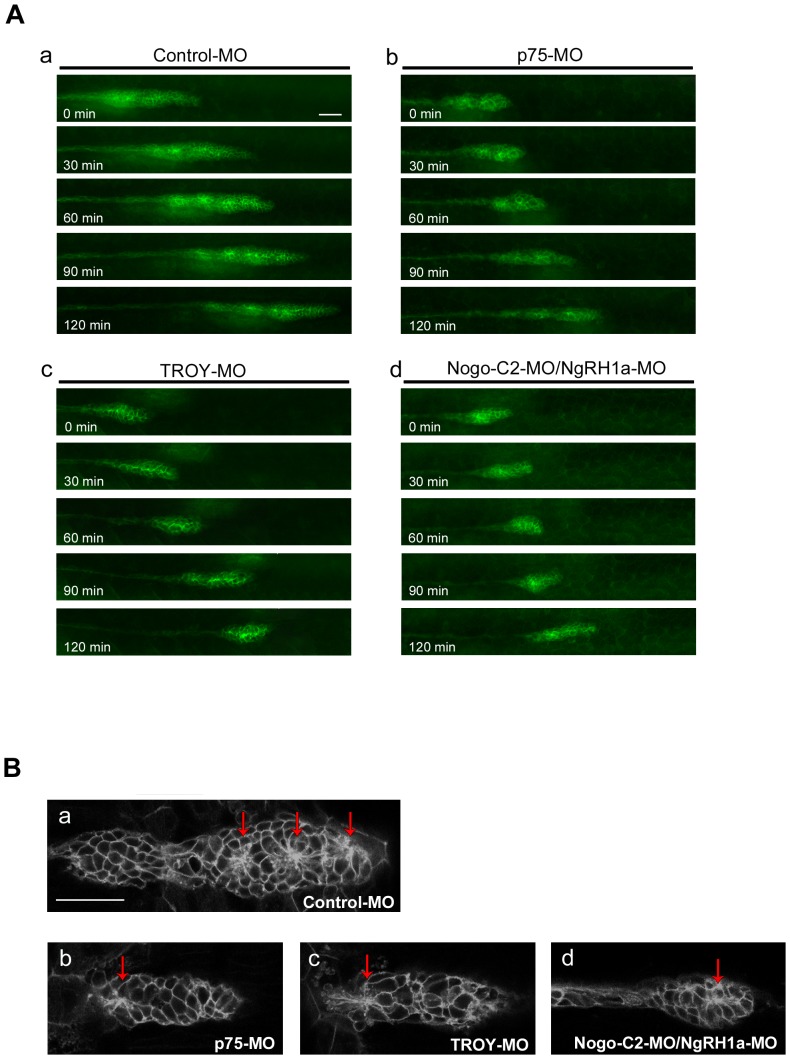
Knockdown of *p75*, *TROY*, or *Nogo-C2*/*NgRH1a* resulted in disordered morphogenesis of the PLL primordium during caudal migration. (A) Transgenic CldnB::lynEGFP zebrafish were injected with control MO (panel a), or MOs against zebrafish *p75* (panel b), *TROY* (panel c), or *Nogo-C2*/*NgRH1a* (panel d), and time-lapse recording was then performed from 28 hpf. The morphology of PLL primordium in the morphants was unstable during migration, as shown at 30-min intervals (panels b–d). (B) Rosette formation in control (panel a), *p75* (panel b), *TROY* (panel c), and *Nogo-C2*/*NgRH1a* (panel d) morphants. Rosettes are indicated by red arrows. Scale bar, 20 µm.

We have demonstrated that injection of zebrafish embryos with MOs against *p75*, *TROY*, *Nogo-C2*, and *NgRH1a* results in a prominent reduction in neuromast number, without affecting proliferation of cells in the PLL primordium (Fig. 2, 3 and 6). We next sought to monitor the status of the PLL primordium during caudal migration through time lapse recording. We injected MOs into CldnB::lynEGFP transgenic line, and then traced the migration of the PLL primordium in each morphant from 28 hpf. As shown in Figure 7A, the morphology of the PLL primordium in each morphant differs from that of control embryos during migration. The primordium of embryos injected with control MO maintained a steady conformation during migration (Fig. 7A, panel a). In contrast, PLL primordium in the morphants exhibited extremely unstable organization, in that their size and shape were different at each of the 30-minute intervals (Fig. 7A, panels b–d). While directional migration of the PLL primordium proceeded normally in *p75*, *TROY* and *Nogo-C2/NgRH1a* morphants (Movie S1, S2, S3, S4), the migration rates were slower as compared with control MO-injected embryos; this suggests that the unstable organization caused by *p75*-, *TROY*-, or *Nogo-C2/NgRH1a*-MO injection may interfere with the collective migration of cells in the PLL primordium.

As rosette formation in the migrating primordium is critical for proneuromast deposition, we next investigated whether rosette formation was also affected in *p75*, *TROY*, and *Nogo-C2/NgRH1a* morphants. Confocal microscopy revealed that while two to three rosettes formed in the PLL primordium of control embryo (Fig. 7B, panel a), the malformed primordium of *p75*, *TROY*, and *Nogo-C2/NgRH1a* morphants contained only one or no proneuromasts, and the cores of the associated rosettes were relatively undefined (Fig. 7B, panels b–d). These data, combined with the observed reduction in neuromasts in the morphants, suggest that the Nogo-C2 complex is required for rosette formation and primordium organization, which in turn enables formation of neuromasts in zebrafish.

### Disruption of the Rtn4-m/Nogo-C2 complex results in reduced expression of downstream markers of Fgf signaling in the PLL primordium

**Figure 8:**
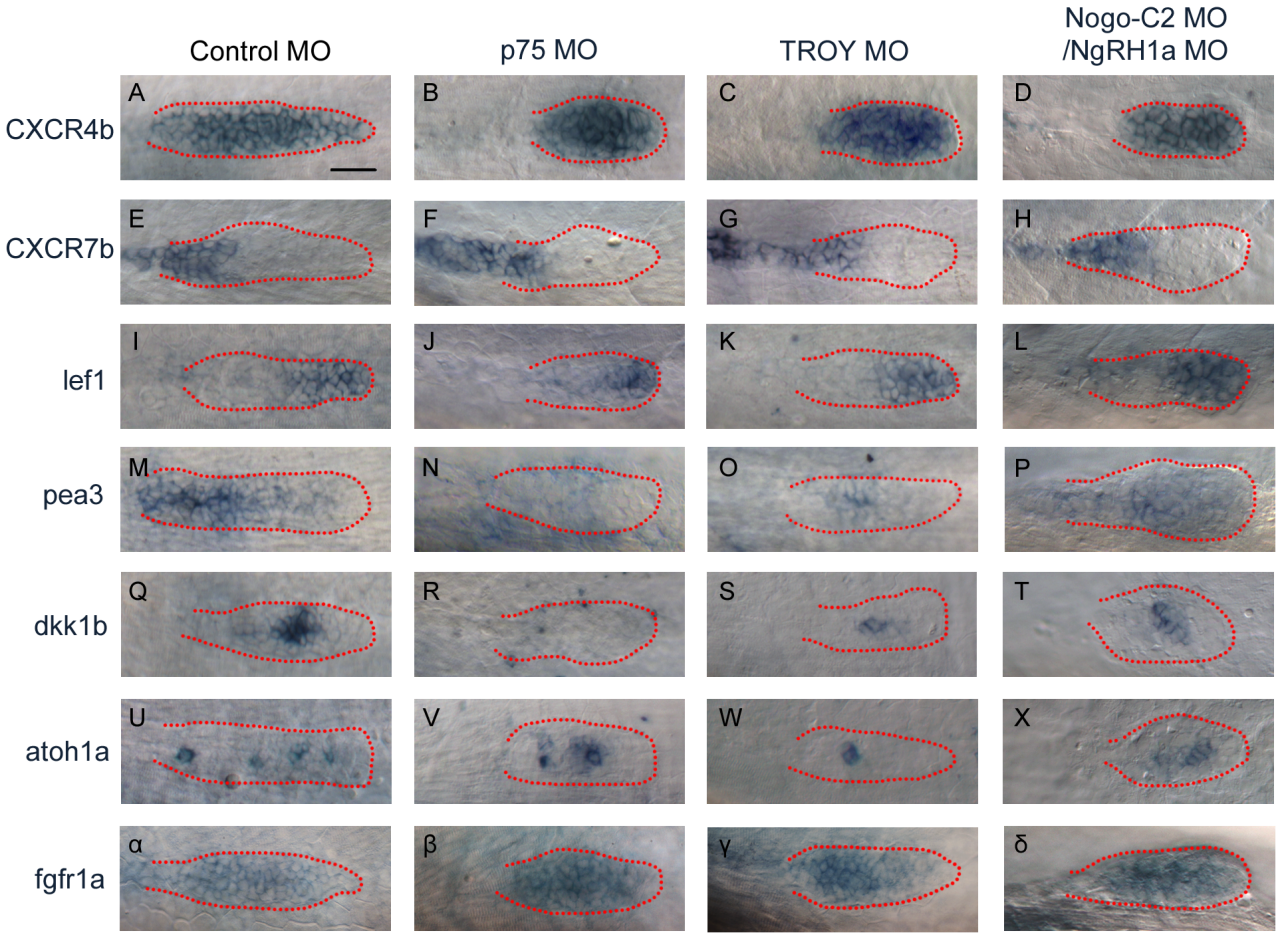
Morphants of *p75*, *TROY*, and *Nogo-C2/NgRH1a* with malformed PLL primordium exhibit decreased expression of downstream markers of Fgf signaling. Whole-mount *in situ* hybridization was performed with probes against markers of PLL primordium organization in control embryo and the indicated morphants. The expression patterns of *cxcr4b* (A–D), *cxcr7b* (E–H), and *lef1* (I–L) were similar in control embryo and morphants. Expression of downstream markers of Fgf signaling (*pea3* (M–P), *dkk1b* (Q–T), and *atoh1a* (U–X)) were reduced in all morphants examined as compared to the control. (α–δ) The expression pattern of *fgfr1a* in the PLL primordium was no different between the control and the morphants. Scale bar, 20 µm.

During the caudal migration of the PLL primordium, crosstalk between Wnt and Fgf signaling is considered to be prerequisite for cell proliferation, primordium morphogenesis, rosette formation, and neuromast deposition [5]. We have demonstrated that MO knockdown of *p75*, *TROY*, or *Nogo-C2*/*NgRH1* affects the size and shape of migrating primordium (Fig. 6 and 7). In addition, rosette formation was affected in *p75*, *TROY*, and *Nogo-C2/NgRH1a* morphants (Fig. 7). We next asked whether knockdown of *p75*, *TROY*, or *Nogo-C2*/*NgRH1* causes neuromast loss by disrupting activation of the Wnt or Fgf pathways in the PLL primordium. We injected embryos of the CldnB::lynEGFP transgenic line with one of the above MOs, and then collected morphants exhibiting aberrant morphogenesis of the PLL primordium at 28–30 hpf; these embryos were used to analyze the expression of downstream markers of Wnt and Fgf signaling involved in primordium migration and integration. To determine whether the leading and trailing zones of the primordium were properly organized during migration, we first analyzed the expression of the chemokine receptor genes *cxcr4b* and *7b* in the PLL primordium. Consistent with the reduction in primordium size, the *cxcr4b* expression region in the *p75*, *TROY*, and *Nogo-C2*/*NgRH1a* morphants was much smaller than that in control embryos; however, the relative expression patterns of *cxcr4b* and *cxcr7b* were essentially unaffected in migrating PLL primordium (Fig. 8A–H). We next investigated the expression of representative downstream markers of Wnt and Fgf signaling in the PLL primordium. As shown in Figures 8I and 8M, Wnt signaling-dependent *lef1* transcripts were detected in the leading zone of the primordium of control embryos; in addition, Fgf signaling-dependent *pea3* transcripts were expressed in the trailing zone. Surprisingly, *lef1* expression was rarely affected in *p75*, *TROY*, and *Nogo-C2/NgRH1a* morphants with disordered primordium (Fig. 8J–L); however, *pea3* transcripts were clearly reduced in the PLL primordium (Fig. 8N–8P). In order to establish whether activation of Fgf signaling was disrupted in the trailing zone of the PLL primordium, we examined other downstream markers of Fgf signaling. Notably, expression of *dkk1b* (Fgf signaling-dependent diffusible inhibitor of Wnt signaling in the primordium) was markedly reduced in *p75*, *TROY*, and *Nogo-C2*/*NgRH1a* morphants as compared to controls (Fig. 8Q–T). The *atoh1a* gene is required for rosette formation and polarization, and is expressed in hair-cell progenitors in the PLL primordium (Fig. 8U). Consistent with the observed reduction in rosette formation (Fig. 7B), *atoh1a*-positive cells were also decreased in *p75*, *TROY*, and *Nogo-C2*/*NgRH1a* morphants (Fig. 8V–X). Interestingly, while the expression of markers related to Fgf activation were down-regulated, expression of *fgfr1a* itself was unaffected in the morphants (Fig. 8α–δ). In summary, the Nogo-C2 complex may be involved in Fgf signaling in the PLL primordium.

### Nogo/Nogo receptor signaling regulates neuromast formation by mediating expression of *dkk1b* in zebrafish

**Figure 9:**
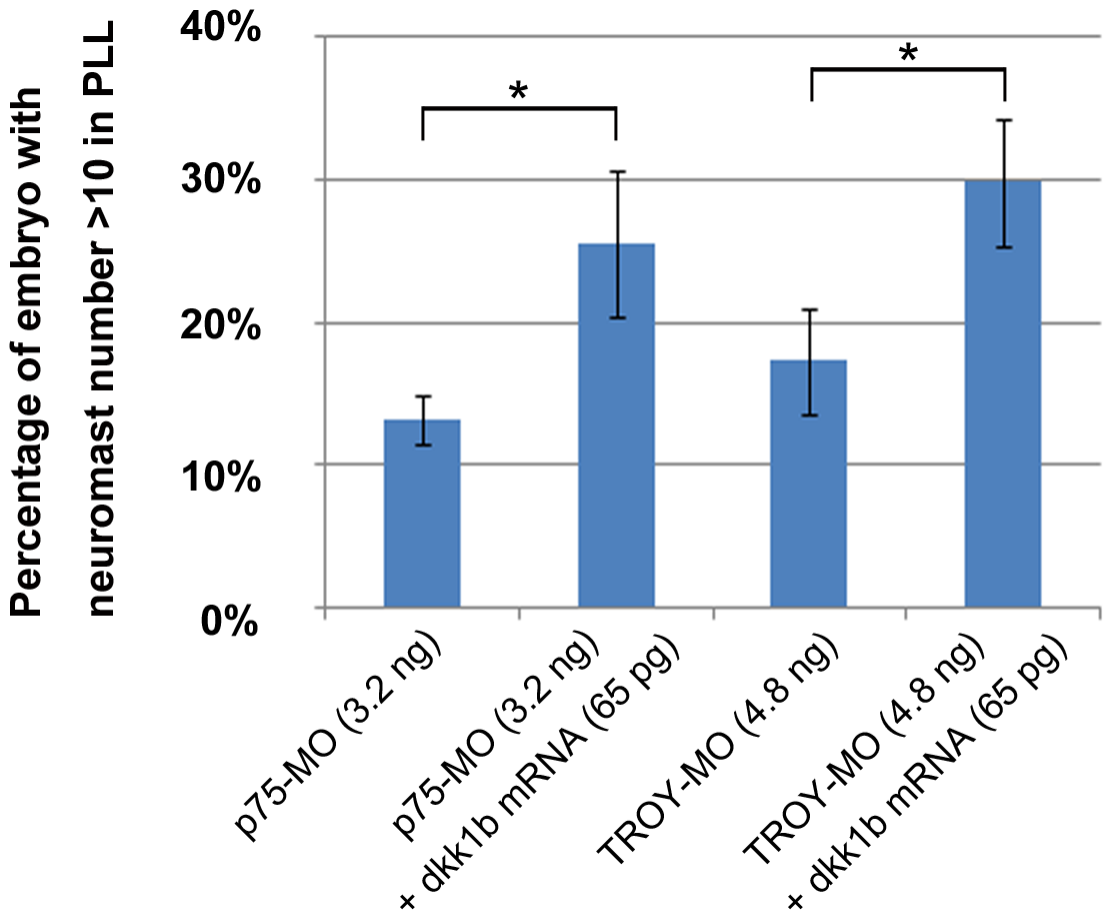
Rescue of neuromast formation in *p75* and *TROY* morphants by injection of *dkk1b* mRNA. Zebrafish embryos were co-injected with *p75*-MO or *TROY*-MO and full-length *dkk1b* mRNA (including the 5′- and 3′-UTR). Neuromast formation in the PLL system was subsequently examined by 4-Di-2-ASP staining at 72 hpf. The percentage of *p75* and *TROY* morphants with neuromast number >10 in PLL system are presented as the mean ± SD and analyzed by unpaired Student's *t*-test from at least three independent experiments. *indicates significant difference compared with the respective controls (P<0.05). MO and *dkk1b* mRNA dosages used are indicated.

We have demonstrated that functional obstruction of Nogo-C2 complex disrupts formation of the PLL system and decreases expression of *dkk1b* (Fig. 2, 3, 7, and 8). We thus suggest that neuromast loss in *p75*, *TROY*, *Nogo-C2*, and *NgRH1a* morphants is related to reduced expression of *dkk1b* in the PLL primordium. In order to test this hypothesis, we attempted to rescue the developmental defects of the PLL in the morphants through ectopic expression of full-length *dkk1b* mRNA. We co-injected zebrafish embryos with *dkk1*b mRNA (coding region only) and either *p75*- and *TROY*-MO; however, severe defects in notochord and somite formation were observed in many embryos, as previously reported [34], preventing PLL development from being studied in these embryos. To regulate translation of exogenous *dkk1b* mRNA, we performed rescue experiments with full-length zebrafish *dkk1b* mRNA flanked by the 5′- and 3′-UTR. Interestingly, the defects in notochord and somite formation were prevented, and neuromast formation in *p75* and *TROY* morphants was partially rescued (Fig. 9); this indicates that Nogo/Nogo receptor signaling modulates the expression of *dkk1b*, which contributes to stable organization of the PLL primordium and neuromast formation in zebrafish.

## Discussion

Accumulating evidence is uncovering the genes and signal pathways that participate in neuromast formation and differentiation. Nogo receptor signaling has previously been shown to be required for PLL neurite outgrowth in zebrafish [27]. In addition, the PLL ganglion and PLL primordium originate from the same cephalic placode, and migration of the PLL primordium is accompanied by the growth cone of the PLL neurite [28], [35], [36], prompting us to search for additional roles of Nogo receptor signaling in PLL development in zebrafish. Based on our findings, we now propose that Nogo-C2, NgRH1a, p75, and TROY form a novel Nogo-C2/Nogo receptor complex, which regulates the expression of *dkk1b* to facilitate neuromast formation in zebrafish.

It is well-known that migration of the PLL primordium is guided by SDF1a/CXCR4b/CXCR7b signaling. Functional disruption of SDF1a or CXCR4b results in disruption of primordium migration and loss of PLL neuromasts. Morpholino knockdown of SDF1a or CXCR4b stalls the migration of the PLL primordium, and similar defects are observed in *cxcr4b* and *sdf1a* mutants [33], [37]. We have demonstrated that knockdown of *Nogo-C2*, *NgRH1a*, *p75*, or *TROY* reduces PLL neuromasts in zebrafish embryos. However, terminal neuromasts of the PLL system still formed at 72 hpf, indicating that the migration of the PLL primordium was not affected in *Nogo-C2*, *NgRH1a*, *p75*, or *TROY* morphants. By using the CldnB::lynEGFP transgenic line, we confirmed that directional migration of the PLL primordium was unaffected in the morphants; these results were also consistent with the findings that *cxcr4b* and *7b* expression were similar between morphant and control embryos. It has been reported that the disappearance of rosettes caused by SU5402 treatment can re-assemble after removal of the inhibitor, indicating PLL primordium can be self re-organized. In addition, this earlier study also demonstrated that the migration rate of the PLL primordium is decreased during the re-organization period [9]. In this study, we used the time-lapse recording of PLL primordium migration in *p75*, *TROY*, and *Nogo-C2*/*NgRH1a* morphants as well as control MO-injected embryos (Fig. 7). We can observe that the structure of PLL primordium in all morphants was changed dynamically with altered pattern or smaller size compared to control MO-injected embryos. Thus, the migration rate of the PLL primordium in all morphants is decreased compared to control MO-injected embryos (Fig. 7).

Wnt signaling participates in many processes during embryonic development and cancer progression; it exerts its effects through activation of the β-catenin/TCF transcription complex and expression of downstream genes, such as CyclinD1 and Axin2 [38], [39]. Like other signaling pathways, Wnt signaling contains a feedback loop; it is negatively regulated by DKK1, the expression of which is controlled by the β-catenin/TCF transcription complex [40]. DKK1 plays important roles in development, especially in head induction [41]. Mutation of murine DKK1 disrupts head formation and limb morphogenesis [42]. In addition, it has been reported that DKK1 regulates the differentiation of dopaminergic neurons in the ventral midbrain, and the development of hair follicles [43], [44]. In zebrafish, DKK1 is required for specification of the anterior neural system and formation of the axial mesendoderm. Overexpression of DKK1 in zebrafish enlarges the head and disrupts notochord and somite formation [34], [45].

During PLL development, Wnt signaling is activated in the leading zone of the primordium, where it participates in cell migration and proliferation [6], [8]. In order to maintain appropriate activation of Wnt signaling in the PLL primordium, *dkk1b* is expressed in its mid-region [6]. Several reports have demonstrated that defects in PLL development result from *dkk1b* overexpression. Forced expression of *dkk1b* through use of the HSP promoter totally abolished the expression of markers of the PLL primordium, including *fgf3*, *fgf10a*, *pea3*, *sef*, and *atoh1a*, leading to defects in cell proliferation, neuromast deposition, and other processes [6], [7]. MO knockdown of *dkk1b* results in enlargement of the expression regions of *lef1* and *axin2* in the PLL primordium, indicating over-activation of Wnt signaling [6]. Here, we demonstrate that functional disruption of the Nogo-C2 complex results in down-regulation of *dkk1b* expression in the PLL primordium. However, the expression region of *lef1* and cell proliferation in the PLL primordium were unaffected, indicating that Wnt signaling activation in the PLL primordium may be restricted by some other mechanism under our experimental conditions. Zebrafish contains a homologue of *dkk1*, called *dkk1a* [46]. We did not observe expression of *dkk1a* in the PLL primordium (data not shown), ruling out a role for *dkk1a* in inhibiting Wnt signaling in the PLL primordium.

TROY expression has been recently reported to be regulated by canonical Wnt signaling, which is required for the control of angiogenesis and blood-brain barrier formation in zebrafish [30]. Furthermore, TROY has been identified as a downstream target of Wnt signaling in human colorectal cancer cell lines. Knockdown of β-catenin using siRNA diminishes expression of TROY in these cells [47]. Thus, if TROY is downstream of Wnt signaling in the PLL primordium, this may explain why *lef* expression was unaffected in the PLL primordium of TROY morphants.

As mentioned above, Wnt activity was not abolished in p75, TROY, or *Nogo-C2*/*NgRH1a* morphants, suggesting that Fgf3 and FgF10a were unaffected as well. We demonstrated that *fgfr1a* was still expressed in the trailing zone of the PLL primordium; however, several important downstream markers of Fgf exhibited disrupted expression, indicating that Nogo-C2 complex signaling may regulate activity downstream of Fgf in the PLL primordium. Trailing cells of the PLL primordium secrete *dkk1b* when Fgf signaling is active during migration. Treatment with the Fgf signaling inhibitor SU5402 abolishes expression of *dkk1b* in the PLL primordium [6]. We demonstrated that injection of p75 and TROY morphants with *dkk1b* mRNA rescued neuromast formation (Fig. 9). Thus, we propose that crosstalk between Nogo and Fgf signaling is essential for *dkk1b* expression in the PLL primordium. As shown in Movie S3, we can observe that the structure of PLL primordium in TROY morphants was changed dynamically, either altered pattern or similar to that in control embryos. To confirm that the reduced expression of dkk1b destabilizes PLL primordium organization in both conditions, we thus analyzed the expression of *dkk1b* in TROY morphants which showed PLL primordium structure similar to that in control embryos. We observed that *dkk1b* expression was still markedly reduced in these morphant (data not shown). This is strong evidence in support of our proposal that decreased expression of *dkk1b* destabilizes the organization of the PLL primordium during migration, leading to loss of neuromasts from the zebrafish LL.

In conclusion, our findings provide the first evidence for a novel mechanism underlying neuromast formation in zebrafish. PLL neuromasts were decreased in zebrafish embryos injected with MOs against *Nogo-C2*, *NgRH1a*, *p75*, or *TROY*, and disorganized PLL primordium and decreased expression of dkk1b were observed in *p75*, *TROY*, and *Nogo-C2*/*NgRH1a* morphants. We propose regulation of *dkk1b* expression by the Nogo-C2/Nogo receptor complex is required for morphogenesis of the zebrafish lateral line primordium.

## Materials and Methods

### Zebrafish care

Zebrafish embryos were raised at 28.5°C, and developmental stages were determined based on the criteria described in the Zebrafish Book [48]. All animal procedures were approved by Academia Sinica Institutional Animal Care and Utilization Committee (ASIACUC) (protocol #10-12-114).

### Total RNA extraction and first-strand cDNA synthesis

Total RNA was isolated using Trizol reagent (Invitrogen, Carlsbad, CA, USA) according to the manufacturer's instructions. Fifty micrograms of total RNA was subjected to the first-strand cDNA synthesis with oligo-dT and random primers (Promega, Madison, WI, USA) using Superscript II RT (Invitrogen, Carlsbad, CA, USA).

### Isolation of full-length *p75*, *LINGO-1*, and *TROY* from zebrafish

Complementary (c)DNAs encoding the complete ORF of zebrafish *p75*, *LINGO-1*, and *TROY* were obtained by PCR amplification with the following gene-specific primers: p75 forward primer, 5′-TCC AGC ATG TTC ACT GTG ACT GTA G-3′ and reverse primer, 5′-GAC TTC TCA TAC GGC AGA TGT GGC-3′; LINGO-1 forward primer, 5′-ATG GTG GCC AGA GAG GCA AGT GGG CAC AGC-3′ and reverse primer, 5′-TTA TAT CAT CTT CAT ACT GAT CTT GTG-3′; and TROY forward primer, 5′-ATG CCG CAG GCT CAG ATC CTC TCC-3′ and reverse primer, 5′-TCA TCC TTC AGT GTC AGT GGT CTG-3′.

### Whole-mount *in situ* hybridization

The zebrafish cDNAs of *p75*, *TROY*, and *LINGO-1* were individually subcloned into pGEM-T Easy (Promega, Madison, WI, USA) for RNA probe synthesis. In addition, the following antisense RNA probes were used: *eya1* [49], *lef1* [50], *pea3* [51], *dkk1b* [6], *atoh1a* [11], *fgfr1a* [9], *cxcr4b* and *cxcr7b* [3]. Digoxigenin-labeled antisense RNA probes were generated by *in vitro* transcription using T7 or SP6 RNA polymerase (Promega, Madison, WI, USA). Whole-mount *in situ* hybridization was performed as previously described [52].

### Immunoprecipitation

Monkey kidney fibroblast COS-1 cells (ATCC CRL-1650; Manassas, VA, USA) were cultured in high-glucose Dulbecco's modified Eagle's medium (DMEM), supplemented with 10% fetal bovine serum (FBS; Hyclone, Logan, UT, USA) in a humidified atmosphere of 5% CO_2_ at 37°C.

Plasmids overexpressing p75-Myc and/or TROY-FLAG fusion proteins were transfected into 70% confluent COS-1 cells using the PolyJet In Vitro DNA Transfection Reagent (SignaGen Laboratories, Ijamsville, MD, USA) in accordance with the manufacturer's instructions. At 48 h post-transfection, one 10-cm dish of COS-1 cells was washed twice with ice-cold PBS. Cells were lysed in 0.4 ml immunoprecipitation lysis buffer (150 mM NaCl, 20 mM HEPES (pH 7.2), 10 mM NaF, 1 mM EDTA, 0.5% NP-40, 1 mM Na_3_VO_4_, 1 mM PMSF, and 1 mM DTT), and then incubated for 30 min at 4°C. The lysate was centrifuged at 14000 rpm for 10 min. One milligram of total protein from the supernatant was pre-cleared with protein A/G Sepharose beads (Santa Cruz Technology, Santa Cruz, CA, USA), and then incubated with 1 µg of monoclonal anti-c-Myc antibody (Sigma) at 4°C overnight. The protein complex was immunoprecipitated with the addition of protein A/G Sepharose beads. Beads were washed 5 times with ice-cold lysis buffer, and the bound proteins were released and denatured by boiling in protein sample buffer for 5 min. The immunoprecipitated proteins were analyzed by Western blotting with the following antibodies: mouse anti-c-Myc (Sigma), mouse anti-FLAG M2 (Sigma), and HRP-conjugated TrueBlot™ anti-mouse-IgG (eBiosciences, San Diego, CA, USA).

### Capped mRNA synthesis

Full-length cDNA fragments of zebrafish *dkk1b* (NM_131003) with or without the untranslated region (UTR) were amplified and inserted into the T7TS vector. T7TS plasmids containing zebrafish *dkk1b* cDNA were linearized with *Xba*I. Capped *dkk1b* mRNA was transcribed from 1 ug linearized plasmid using the mMESSAGE mMACHINE T7 Kit (Ambion, Foster City, CA, USA).

### Injection of morpholinos and capped mRNA

Antisense morpholino oligonucleotides (MOs) against zebrafish p75, LINGO-1, TROY, NgR, NgRH1a, NgRH1b, NgRH2a, Rtn4-l/Nogo-B, Rtn4-m/Nogo-C2, and Rtn4-n/Nogo-C1, and a control MO, were obtained from Gene Tools (Philomath, OR, USA). The sequence of each MO is shown in Table 1.

**Table 1:** Sequences of all morpholino oligonucleotides used in this study.

Gene	Morpholino oligonucleotide sequence
Nogo-B	5′-TCCATTGTTGCGAATGTGTCGACAG-3′
Nogo-C1	5′-AATCCATCTCAGCTCATCTGCGATC-3′
Nogo-C2	5′-GGTGTTATCTGAATTGGCGTGCATC-3′
NgR	5′-GTGTTCTCCAGTTCAACGGGGACCT-3′
NgRH1a	5′-CCGAGAGCGAGTTAGCCTGAGGAGT-3′
NgRH1b	5′-TCCGCGCAGCCGCACGAGTTGTCAT-3′
NgRH2a	5′-GTATTCCAGACTTCACACTGTCAGT-3′
p75	5′-AAGACGGTGGTCCTGGATGAAGTAC-3′
LINGO-1	5′-CATTCTGCCGGAGTGCAGCTTAGAT-3′
TROY	5′-TCTCCGGTGATCTGCGCTCAGTAAG-3′
Control	5′- CCTCTTACCTCAGTTACAATTTATA-3′

MO and capped mRNA were injected into wild-type zebrafish embryos using a microinjection system consisting of a SZX9 stereomicroscope (Olympus, Tokyo, Japan) and an IM300 Microinjector (Narishige, Tokyo, Japan). The capped mRNA was injected into embryos at the 1-cell stage, while MOs were injected at the 2- to 4-cell stage.

### Neuromast staining

The hair cells of neuromasts in live zebrafish embryos were stained with the fluorescent dye 4-(4-(diethylamino)styryl)-*N*-methylpyridinium iodide (4-Di-2-ASP) (Molecular Probes, Eugene, OR, USA) at 72 hpf, as described before [29]. Images were captured using an Olympus 1X70 fluorescence microscope (Tokyo, Japan) with a SPOT camera (Diagnostic Instruments, Sterling Heights, MI, USA).

### Alkaline phosphatase (AP)-binding assay

To generate AP fusion proteins, N-terminal fragments of zebrafish Nogo-related proteins were amplified and ligated into the APtag-4 vector (a gift from Dr. H. J. Cheng). These expression plasmids were transfected into 70% confluent COS-1 cells, and the secreted AP-fusion proteins were harvested at 3 days post-transfection. The AP binding assay was performed by first transfecting COS-1 cells with one of the following plasmids: pcDNA3, pcDNA3-zNgR, pcDNA3-zNgRH1a, pcDNA3-zNgRH1b, or pcDNA3-zNgRH2a. At 48 h post-transfection, cells were washed twice with HBH (Hank's balanced salt buffer containing 1 mg/mL bovine serum albumin (BSA), and 20 mM HEPES; pH 7.0), and then incubated with conditioned media containing various AP-fusion proteins at room temperature for 3 h. After fixation with 4% paraformaldehyde (PFA) and heat inactivation of endogenous AP at 65°C for 2 h, the bound AP fusion proteins were detected directly by NBT/BCIP (Roche, Mannheim, Germany).

### Expression plasmid construction

To construct the appropriate fusion protein, zebrafish cDNAs (encoding NgR, NgRH1a, NgRH1b, NgRH2a, p75, or TROY) were ligated into the relevant vector (pcDNA3, pcDNA3-Myc-HisA, or pcDNA3-FLAG) (Invitrogen, Carlsbad, CA, USA). The pcDNA3-FLAG vector was created by inserting the DNA fragment encoding the FLAG tag into the pcDNA3 vector. The NgR, NgRH1a, NgRH1b, and NgRH2a cDNAs were obtained by PCR amplification, based on the following entries in the NCBI GenBank database: accession no.: NM_203478, NM_203479, NM_203483, and NM_203480, respectively.

### Time-lapse recording and confocal images

For time-lapse recording, zebrafish embryos were anesthetized in 0.02% tricaine (Sigma), and then embedded in 1.5% low-melting point agarose. Time-lapse images were taken at 30-minute intervals over 15 hours with z-stack collection using a Leica TCS SP5 X confocal microscope; the images were then processed using ImageJ software. High resolution confocal images of the PLL primordium were captured using a Zeiss LSM510 laser scanning confocal microscope (Carl Zeiss, Jena, Germany).

### BrdU incorporation assay and whole-mount immunostaining

BrdU incorporation assays were performed from 32.5 hpf. Briefly, dechorionated zebrafish embryos were incubated in 15% DMSO fish water containing 10 mM BrdU (Sigma) for 30 minutes at 4°C, and then incubated at 28°C for one hour; embryos were subsequently fixed in PFA at 4°C overnight for immunostaining.

Whole-mount immunostaining was performed following standard protocols as previously described [53] with some modifications. The antibodies used were as follows: mouse anti-BrdU (Sigma), rabbit anti-GFP (Cell Signaling Technology, Inc., MA), Cy2-conjugated anti-mouse IgG, and Cy3-conjugated anti-rabbit IgG (Jackson ImmunoResearch Laboratories, Inc., West Grove, PA). The nuclei were visualized by staining with DAPI (Sigma) for 5 minutes.

### TUNEL assay

For detection of apoptotic cells, the embryos were fixed in 4% PFA overnight, washed several times with PBST, and stored in methanol at −20°C. To perform TUNEL assay, embryos were rehydrated with methanol/PBST series, treated with proteinase K, and then fixed in 4% PFA at room temperature. Apoptotic cells were detected by In Situ Cell Death Detection Kit (Roche Diagnostics, Germany) according to instructions of the manufacturer.

## Supporting Information

Figure S1 **Amino acid sequence alignments of zebrafish, human, and mouse Nogo co-receptor proteins.** Identical amino acids are shown in white on a black background. The signal peptide and transmembrane domain are underlined. (A) Alignment of zebrafish, human, and mouse p75 amino acid sequences, as generated using CLUSTAL X. The four cysteine-rich domains (CRDs) and the death domain of p75 are indicated. (B) Alignment of zebrafish, human, and mouse LINGO-1 amino acid sequences. The leucine-rich repeat (LRR) motif and immunoglobulin (Ig)-like domain are indicated. (C) Alignment of zebrafish, human, and mouse TROY amino acid sequences. The three CRDs in the extracellular region are indicated.
(PDF)

Figure S2 **Control experiments for morpholino specificity.** To confirm the specificities of the MOs against p75, LINGO-1, and TROY, pCMV-GFP reporter plasmids containing a perfect or mismatched MO target sequence were generated. The pCMV-GFP reporter plasmids bearing the perfect MO target sequence (mo-GFP) were injected into zebrafish embryos either alone (A–C) or together with the relevant MO (A′–C′). As controls, zebrafish embryos were co-injected with MO and pCMV-GFP reporter plasmid containing the mismatched target sequence (MM mo-GFP) (A″–C″). All images were taken from zebrafish embryos at 48 hpf.
(PDF)

Figure S3 **ATG-MOs reduced the protein levels of p75 and TROY, and splice-blocking MOs also affected PLL development.** (A–B) Total proteins from (A) p75 and (B) TROY morphant embryos were collected at 24 hpf and subjected to Western blotting with anti-p75 (abcam, ab32888) or anti-TROY (Enzo, ALX-210-801) antibodies, as indicated. GADPH was used as a loading control. (C–D) Zebrafish embryos were injected with splice-blocking MOs against either (C) p75 or (D) TROY, and the neuromasts were stained with 4-Di-2-ASP at 72 hpf (upper left panels). The numbers of PLL neuromasts in these morphants at 72 hpf are summarized (lower left panels). The MO dosages used and sample numbers (N) are indicated. The efficiency and specificity of p75-SB-MO and TROY-SB-MO were verified by RT-PCR with two primer sets, as illustrated in the top right panels. The sequences of splice-blocking MOs and primers used in RT-PCR are provided.
(PDF)

Figure S4 **Control experiments for morpholino specificity.** To confirm the specificities of the MOs against three Nogo ligands and four Nogo receptors, pCMV-GFP reporter plasmids containing a perfect or mismatched MO target sequence corresponding to that MO were generated. The pCMV-GFP reporter plasmids bearing the perfect MO target sequence (mo-GFP) were injected into zebrafish embryos either alone (A–G) or together with the relevant MO (A′–G′). As controls, zebrafish embryos were co-injected with MO and pCMV-GFP reporter plasmid containing the mismatched target sequence (MM mo-GFP) (A″–G″). All images were taken from zebrafish embryos at 48 hpf.
(PDF)

Figure S5 **Splice-blocking MO against Nogo-C2 and NgRH1a also disrupted PLL development in zebrafish.** Zebrafish embryos were injected with splice-blocking MOs against either (A) Nogo-C2 or (B) NgRH1a, and the neuromasts were stained with 4-Di-2-ASP at 72 hpf (upper left panels). The numbers of PLL neuromasts in these morphants at 72 hpf are summarized (lower left panels). The MO dosages used and sample numbers (N) are indicated. The efficiency and specificity of Nogo-C2-SB-MO and NgRH1a-SB-MO were confirmed by RT-PCR with two primer sets, as illustrated in the top right panels. The sequences of splice-blocking MOs and primers used in RT-PCR are provided.
(PDF)

Figure S6 **Non-specific MO-induced apoptosis was not observed in** ***p75*** **,** ***TROY*** **and** ***Nogo-C2/NgRH1a*** **morphants.** Each MO was injected into the CldnB::lynEGFP transgenic line, and then collected the morphants with malformed PLL primordium at 26–34 hpf for TUNEL assay. One embryo at 30 hpf, representative of a large sample (n = 10), was shown. Apoptotic cells in PLL primordium in control embryos (panels A–A′″), and *p75* (B–B′″), *TROY* (C–C′″), and *Nogo-C2*/*NgRH1a* (D–D′″) morphants were revealed by TUNEL staining in red. Nuclei were also stained with DAPI. Scale bar, 20 µm.
(PDF)

Figure S7 **The mRNAs of p75, TROY and NgRH1a were expressed in the migrating primordium in zebrafish.** Whole-mount *in situ* hybridization was performed with antisense probes against *p75* (A), *TROY* (B), and *NgRH1a* (C) in CldnB::lynEGFP zebrafish at 30 hpf. The PLL primordium was simultaneously revealed by immunostaining with anti-GFP antibody (A′–C′). The PLL primordium is labeled with red dots.
(PDF)

Movie S1 **Time-lapse recording of caudal migration of PLL primordium in control MO-injected embryo.** Control MO-injected zebrafish was anesthetized and then embedded in 1.5% low-melting point agarose, and time-lapse recording was started at 28 hpf. The movie showed the lateral line primordium migrating on the body surface of control MO-injected embryo.
(MPG)

Movie S2 **Time-lapse recording of caudal migration of PLL primordium in p75 morphant.** p75 morphant was anesthetized and then embedded in 1.5% low-melting point agarose, and time-lapse recording was started at 28 hpf. The movie showed the lateral line primordium migrating on the body surface of p75 morphant.
(MPG)

Movie S3 **Time-lapse recording of caudal migration of PLL primordium in TROY morphant.** TROY morphant was anesthetized and then embedded in 1.5% low-melting point agarose, and time-lapse recording was started at 28 hpf. The movie showed the lateral line primordium migrating on the body surface of TROY morphant.
(MPG)

Movie S4 **Time-lapse recording of caudal migration of PLL primordium in Nogo-C2/NgRH1a morphant.** NogoC2/NgRH1a morphant was anesthetized and then embedded in 1.5% low-melting point agarose, and time-lapse recording was started at 28 hpf. The movie showed the lateral line primordium migrating on the body surface of NogoC2/NgRH1a morphant.
(MPG)
