# Supplementary material for: The Nogo-C2/Nogo Receptor Complex Regulates the Morphogenesis of Zebrafish Lateral Line Primordium through Modulating the Expression of dkk1b, a Wnt Signal Inhibitor
Source: PLoS One. 2014 Jan 21;9(1):e86345. doi: 10.1371/journal.pone.0086345 (PMC3897714; doi:10.1371/journal.pone.0086345)
Supplement: Figure S1 — Amino acid sequence alignments of zebrafish, human, and mouse Nogo co-receptor proteins. Identical amino acids are shown in white on a black background. The signal peptide and transmembrane domain are underlined. (A) Alignment of zebrafish, human, and mouse p75 amino acid sequences, as generated using CLUSTAL X. The four cysteine-rich domains (CRDs) and the death domain of p75 are indicated. (B) Alignment of zebrafish, human, and mouse LINGO-1 amino acid sequences. The leucine-rich repeat (LRR) motif and immunoglobulin (Ig)-like domain are indicated. (C) Alignment of zebrafish, human, and mouse TROY amino acid sequences. The three CRDs in the extracellular region are indicated. (PDF) [file pone.0086345.s001.pdf]

A

```
zp75 : ----------MFTVT---VVFLLVGEALS-SRDTCPSGAFTTRGECCKQCQPGEGMVKPCGATQTVCEPCLDSETFSENFSHKDKCLPCTQCSEIMRML :  85
hp75 : MG-AGATGRAMDGPR--LLLLLLLGVSLGGAKEACPTGLYTHSGECCKACNLGEGVAQPCGANQTVCEPCLDSVTFSDVVSATEPCKPCTECVGLQSMS :  96
mp75 : MRRAGAACSAMDRLRLLLLLLLLGVSFGGAKETCSTGMYTHSGECCKACNLGEGVAQPCGANQTVCEPCLDSVTFSDVVSATEPCKPCTECLGLQSMS :  99
```

Signal peptide — CRD 1 — CRD 2

```
zp75 : TPCTDANDALCVCDYGFFLNQLSGRCEACTVCPVGQGVQVPCNFERDTVCEKCEDDTYSDQDTSFDPCLPCTVCDQPEMEVKSCTPVSDAVCQDFDAPN : 184
hp75 : APCVEADDAVCRCAYGYYQDETTGRCEACRVCEAGSGLVFSCQDKQNTVCEECPDGTYSDEANHVDPCLPCTVCEDTERQLRECTRWADAECEEIPGRW : 195
mp75 : APCVEADDAVCRCSYGYYQDEETGRCEACSVCGVGSGLVFSCQDKQNTVCEECPEGTYSDEANHVDPCLPCTVCEDTERQLRECTPWADAECEEIPGRW : 198
```

CRD 2 — CRD 3 — CRD 4

```
zp75 : SSPSPSAPPPTPYFTPSPSIHKFSSPGPSVVSYTEKLLPDNPTESVTTTDPGQKRLHGLSDNLIPIYTSILAAVLLGLVAFIIFKRWNSCKQNKQANNR : 283
hp75 : ITRS---TPPEGSDSTAPSTQEPEAP--PEQDLIASTVAGVVTTVMGSSQPVVTR--GTTDNLIPVYCSILAAVVVGLVAYIAFKRWNSCKQNKQGAN- : 286
mp75 : ITRS---TPPEGSDVTTPSTQEPEAP--PERDLIASTVADTVTTVMGSSQPVVTR--GTADNLIPVYCSILAAVVVGLVAYIAFKRWNSCKQNKQGAN- : 289
```

transmembrane domain

```
zp75 : ACSANPSQTPSPEGEKLHSDSGISVDSQSLQDGQGPPHTV----VKIDGGSALSLPLHTREEVEKLLNRTNEGEESAANEETDWCSLAGLLGYKEEHIA : 378
hp75 : --SRPVNQTPPPEGEKLHSDSGISVDSQSLHDQQPHTQTASGQALKGDGGLYSSLPPAKREEVEKLLNGS---------AGDTWRHLAGELGYQPEHID : 374
mp75 : --SRPVNQTPPPEGEKLHSDSGISVDSQSLHDQQTHTQTASGQALKGDGNLYSSLPLTKREEVEKLLN------------GDTWRHLAGELGYQPEHID : 374
```

death domain

```
zp75 : NFKQEERPIQALLSHWASQDSANIDTLCTALKKINREDIAQSIIVKPTATSAV : 431   Identity   Similarity
hp75 : SFTHEACPVRALLASWATQDSATLDALLAALRRIQRADLVESLCSESTATSPV : 427     45%         61%
mp75 : SFTHEACPVRALLASWGAQDSATLDALLAALRRIQRADIVESLCSESTATSPV : 427     45%         61%
```

death domain

B

```
zLINGO-1 : MTFLQVTIKMVAR-EASGHSYLVACWQPILIIMLGTVLSGSATGCPSRCECSAQERSVVCHRRKLITLPEGIPIDTRLLDLSKNRL :  85
hLINGO-1 : ---MQVSKRMLAGGVRSMPSPLLACWQPILLLVLGSVLSGSATGCPPRCECSAQDRAVLCHRKRFVAVPEGIPTETRLLDLGKNRI :  83
mLINGO-1 : ---------MLAGGMRSMPSPLLACWQPILLLVLGSVLSGSATGCPPRCECSAQDRAVLCHRKRFVAVPEGIPTETRLLDLGKNRI :  77
```

Signal peptide — LRR N-cap — LRR

```
zLINGO-1 : KAINPEEFLNYPQLEDLQLNENIISVIEPGAFSNLLGLRTLGLRNNNLKLIQLGVFTGLSNLTRLDISENKIVILLDYMFQELYNL : 171
hLINGO-1 : KTLNQDEFASFPHLEELELNENIVSAVEPGAFNNLFNLRTLGLRSNRLKLIPLGVFTGLSNLTKLDISENKIVILLDYMFQDLYNL : 169
mLINGO-1 : KTLNQDEFASFPHLEELELNENIVSAVEPGAFNNLFNLRTLGLRSNRLKLIPLGVFTGLSNLTKLDISENKIVILLDYMFQDLYNL : 163
```

LRR

```
zLINGO-1 : KELEVGDNDLVFISHRAFHGLSSLEQLTMERCNLTSVPTEAFSHLHNLLTLKLRHLNVNVIRDFSFRRLYRLKILEIANWPLLESL : 257
hLINGO-1 : KSLEVGDNDLVYISHRAFSGLNSLEQLTLEKCNLTSIPTEALSHLHGLIVLRLRHLNINAIRDYSFKRLYRLKVLEISHWPYLDTM : 255
mLINGO-1 : KSLEVGDNDLVYISHRAFSGLNSLEQLTLEKCNLTSIPTEALSHLHGLIVLRLRHLNINAIRDYSFKRLYRLKVLEISHWPYLDTM : 249
```

LRR

```
zLINGO-1 : TAKSLHGLNITTLSITNCNLTAVPYVAIQHLVYLRFFNLSFNPIEVVEGNKMHNLLRLQAFHLVGGRLVSIEPYSFKGLNYLRVLN : 343
hLINGO-1 : TPNCLYGLNLTSLSITHCNLTAVPYLAVRHLVYLRFLNLSYNPISTIEGSMLHELLRLQEIQLVGGQLAVVEPYAFRGLNYLRVLN : 341
mLINGO-1 : TPNCLYGLNLTSLSITHCNLTAVPYLAVRHLVYLRFLNLSYNPIGTIEGSMLHELLRLQEIQLVGGQLAVVEPYAFRGLNYLRVLN : 335
```

LRR

```
zLINGO-1 : VSSNSLSTLEESAFHSVGNLETLALHDNPLACDCRLLWVFRRRWRLNFNRQQPSCETPEFLQGKEFKDFPDVLPPNYFTCQKSKIR : 429
hLINGO-1 : VSGNQLTTLEESVFHSVGNLETLILDSNPLACDCRLLWVFRRRWRLNFNRQQPTCATPEFVQGKEFKDFPDVLLPNYFTCRRARIR : 427
mLINGO-1 : VSGNQLTTLEESAFHSVGNLETLILDSNPLACDCRLLWVFRRRWRLNFNRQQPTCATPEFVQGKEFKDFPDVLLPNYFTCRRAHIR : 421
```

LRR — LRR C-cap

```
zLINGO-1 : DHKAIHRFVDEGTTVQFPCQADGDPTPMIMWQSPKKQFITTKSIGRLSVSLDGTLEVRYAQIQDNGTYTCFAVNAGGNDTRLAHLH : 515
hLINGO-1 : DRKAQQVFVDEGHTVQFVCRADGDPPPAILWLSPRKHLVSAKSNGRLTVFPDGTLEVRYAQVQDNGTYLCIAANAGGNDSMPAHLH : 513
mLINGO-1 : DRKAQQVFVDEGHTVQFVCRADGDPPPAILWLSPRKHLVSAKSNGRLTVFPDGTLEVRYAQVQDNGTYLCIAANAGGNDSMPAHLH : 507
```

Ig domain

```
zLINGO-1 : VHSYSPNWPHQPNKTFAFILNQPSDNSANGTGAMDPFPFDMKTLIIATTMGFISFLGVVLFCLVLLFLWSRGKGNAKPNIEIEYVP : 601
hLINGO-1 : VRSYSPDWPHQPNKTFAFISNQPGEGEANSTRATVPFPFDIKTLIIATTMGFISFLGVVLFCLVLLFLWSRGKGNTKHNIEIEYVP : 599
mLINGO-1 : VRSYSPDWPHQPNKTFAFISNQPGEGEANSTRATVPFPFDIKTLIIATTMGFISFLGVVLFCLVLLFLWSRGKGNTKHNIEIEYVP : 593
```

transmembrane domain

```
zLINGO-1 : RKVDGENSPNEGSHKISMKMI : 622   Identity   Similarity
hLINGO-1 : RKSDAGISSADAPRKFNMKMI : 620     72%         86%
mLINGO-1 : RKSDAGISSADAPRKFNMKMI : 614     71%         85%
```

C

```
zTROY : MPQAQILSRFPLQRILILPLCVLFAVMAEERRDCREQEYKDKFGSCIPCRQCDAGQELSKECGFGYGEDAQCVPCRASRFKEDRSLQKC :  89
hTROY : MALKVLLEQEKTFFTLLVLLGYLSCKVTCESGDCRQQEFRDRSGNCVPCNQCGPGMELSKECGFGYGEDAQCVTCRLHRFKEDWGFQKC :  89
mTROY : MALKVLPLHRTVLFAAILFLLHLACKVSCETGDCRQQEFKDRSGNCVLCKQCGPGMELSKECGFGYGEDAQCVPCRPQRFKEDWGFQKC :  89
```

Signal peptide — CRD 1 — CRD 2

```
zTROY : KPCLDCSLLNRFQKGNCSTTNNAVCGDCLPGFYRKTKLRGFQDMECIPCGDPPPPYETHCMGRVNLVPLPSTVSSPRDMALAAVICSAL : 178
hTROY : KPCLDCAVVNRFQKANCSATSDAICGDCLPGFYRKTKLVGFQDMECVPCGDPPPPYEPHCASKVNLVKIASTASSPRDTALAAVICSAL : 178
mTROY : KPCADCALVNRFQRANCSHTSDAVCGDCLPGFYRKTKLVGFQDMECVPCGDPPPPYEPHCTSKVNLVKISSTVSSPRDTALAAVICSAL : 178
```

CRD 2 — CRD 3

```
zTROY : ATVLLALFILCVIYCKRQLLEKKP-VSMRAHEGPFLGSELSCLDR-RVLELSQRPCCHCTHGSEQTCGGVQLVSSVCCEDVWSQNRRRD : 265
hTROY : ATVLLALLILCVIYCKRQFMEKKPSWSLRSQDIQYNGSELSCFDRPQLHEYAHRACCQCRRDSVQTCGPVRLLPSMCCEEACSPNPATL : 267
mTROY : ATVLLALLILCVIYCKRQFMEKKPSWSLRSQDIQYNGSELSCFDQPRLRHCAHRACCQYHRDSAPMYGPVHLIPSLCCEEARSSARAVL : 267
```

transmembrane domain

```
zTROY : APAFHSHCRISDN-----GRLTNESVGSQTDMACAP-DEVWPLVQ-----------------SRRTTDSLQTNQRCSSCEEDEEEVQIS : 331
hTROY : GCGVHSAASLQARNAGPAGEMVPTFFGSLTQSICGEFSDAWPLMQNPMGGDNISFCDSYPELTGEDIHSLNPELESSTSLDSNSSQDLV : 356
mTROY : GCGLRSPTTLQERNPASVGDTMPAFFGSVSRSICAEFSDAWPLMQNPLGGD-SSLCDSYPELTGEDTNSLNPENESAASLDSSGGQDLA : 355
```

```
zTROY : D-----QRTAEEPEDASHKP-------LIQQTTDTEG---------------------------- : 356   Identity   Similarity
hTROY : GGAVPVQSHSENFTAATDLSRYNNT--LVESASTQDALTMRSQLDQESGAVIHPATQTSLQEA : 417     43%         56%
mTROY : G--TAALESSGNVSESTDSPRHGDTGTVWEQTLAQDAQRTPSQGGWEDRENLNLAMPTAFQDA : 416     42%         56%
```

Figure S1
