# Supplementary figures and images for: The Nogo-C2/Nogo Receptor Complex Regulates the Morphogenesis of Zebrafish Lateral Line Primordium through Modulating the Expression of dkk1b, a Wnt Signal Inhibitor
Source: PLoS One. 2014 Jan 21;9(1):e86345. doi: 10.1371/journal.pone.0086345 (PMC3897714; doi:10.1371/journal.pone.0086345)

Scheme of the pCMV-GFP reporter plasmid.

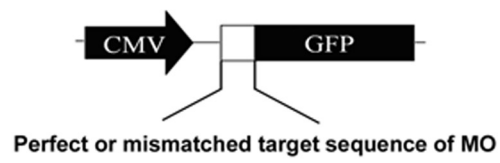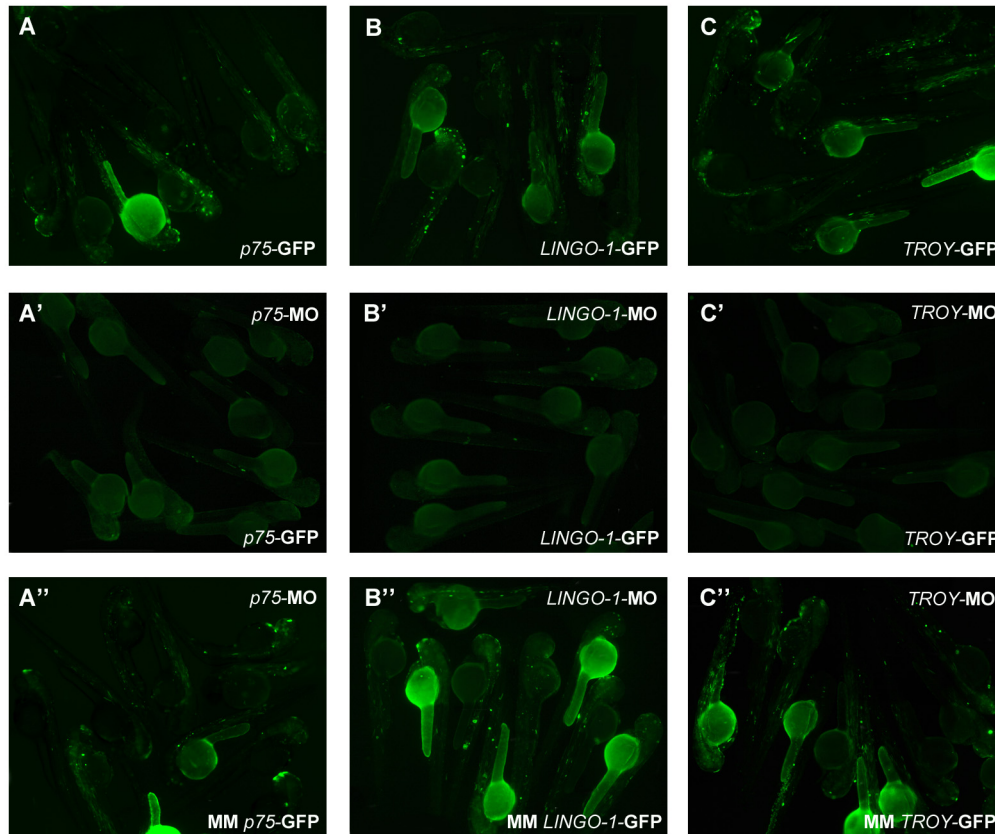

Figure S2

Supplement: Figure S2 — Control experiments for morpholino specificity. To confirm the specificities of the MOs against p75, LINGO-1, and TROY, pCMV-GFP reporter plasmids containing a perfect or mismatched MO target sequence were generated. The pCMV-GFP reporter plasmids bearing the perfect MO target sequence (mo-GFP) were injected into zebrafish embryos either alone (A–C) or together with the relevant MO (A′–C′). As controls, zebrafish embryos were co-injected with MO and pCMV-GFP reporter plasmid containing the mismatched target sequence (MM mo-GFP) (A″–C″). All images were taken from zebrafish embryos at 48 hpf. (PDF) [file pone.0086345.s002.pdf]

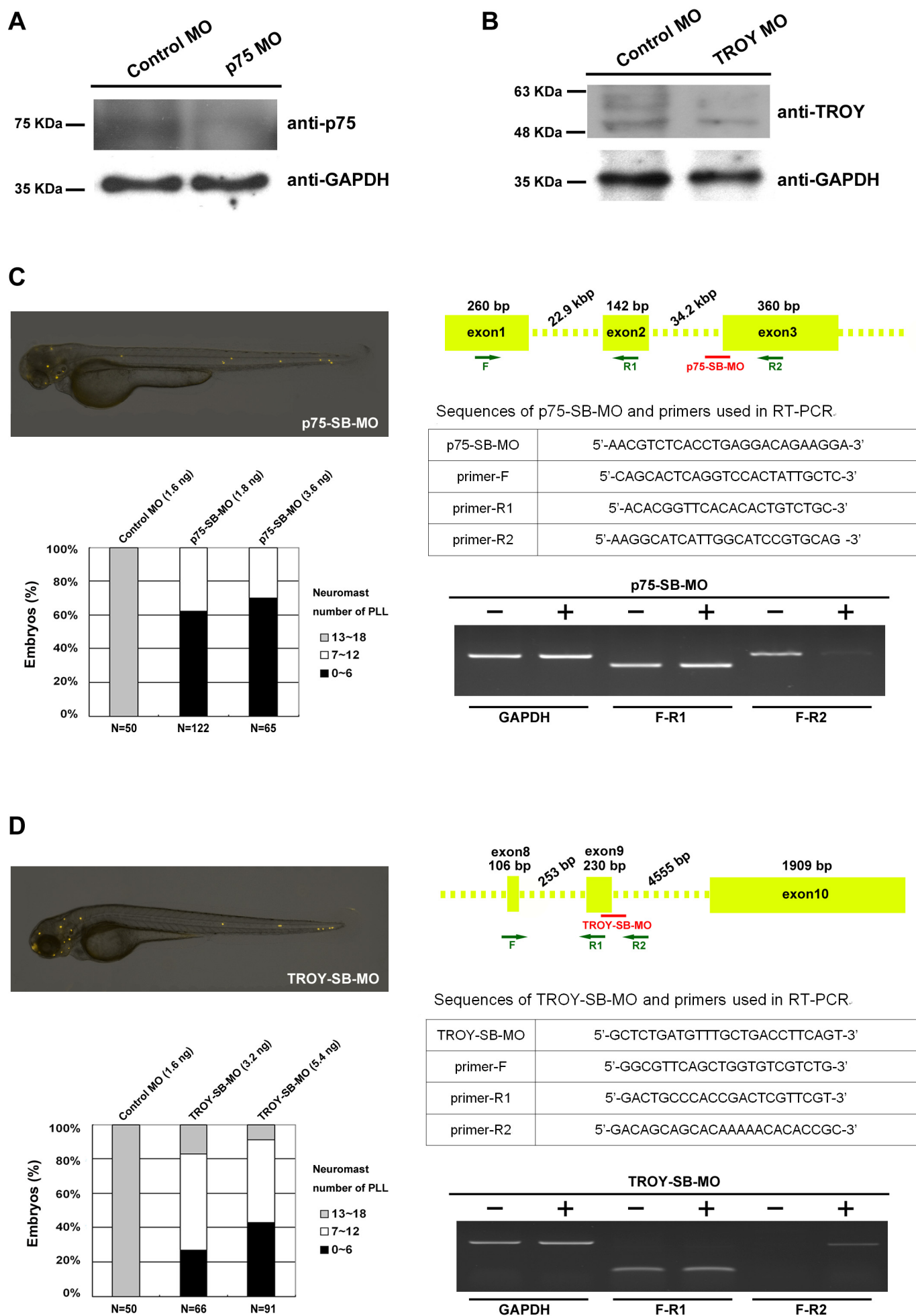

**Figure S3**

Supplement: Figure S3 — ATG-MOs reduced the protein levels of p75 and TROY, and splice-blocking MOs also affected PLL development. (A–B) Total proteins from (A) p75 and (B) TROY morphant embryos were collected at 24 hpf and subjected to Western blotting with anti-p75 (abcam, ab32888) or anti-TROY (Enzo, ALX-210-801) antibodies, as indicated. GADPH was used as a loading control. (C–D) Zebrafish embryos were injected with splice-blocking MOs against either (C) p75 or (D) TROY, and the neuromasts were stained with 4-Di-2-ASP at 72 hpf (upper left panels). The numbers of PLL neuromasts in these morphants at 72 hpf are summarized (lower left panels). The MO dosages used and sample numbers (N) are indicated. The efficiency and specificity of p75-SB-MO and TROY-SB-MO were verified by RT-PCR with two primer sets, as illustrated in the top right panels. The sequences of splice-blocking MOs and primers used in RT-PCR are provided. (PDF) [file pone.0086345.s003.pdf]

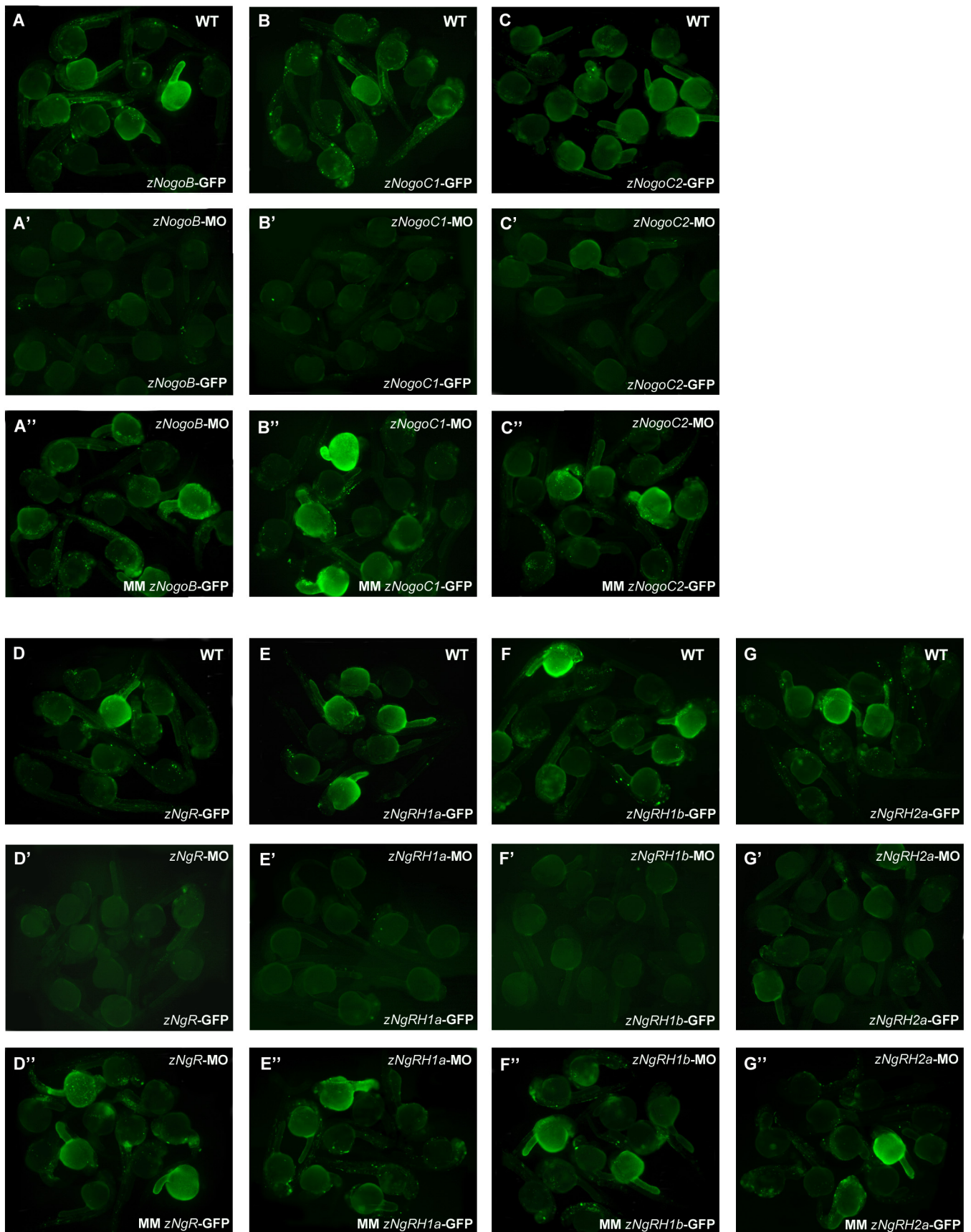

**Figure S4**

Supplement: Figure S4 — Control experiments for morpholino specificity. To confirm the specificities of the MOs against three Nogo ligands and four Nogo receptors, pCMV-GFP reporter plasmids containing a perfect or mismatched MO target sequence corresponding to that MO were generated. The pCMV-GFP reporter plasmids bearing the perfect MO target sequence (mo-GFP) were injected into zebrafish embryos either alone (A–G) or together with the relevant MO (A′–G′). As controls, zebrafish embryos were co-injected with MO and pCMV-GFP reporter plasmid containing the mismatched target sequence (MM mo-GFP) (A″–G″). All images were taken from zebrafish embryos at 48 hpf. (PDF) [file pone.0086345.s004.pdf]

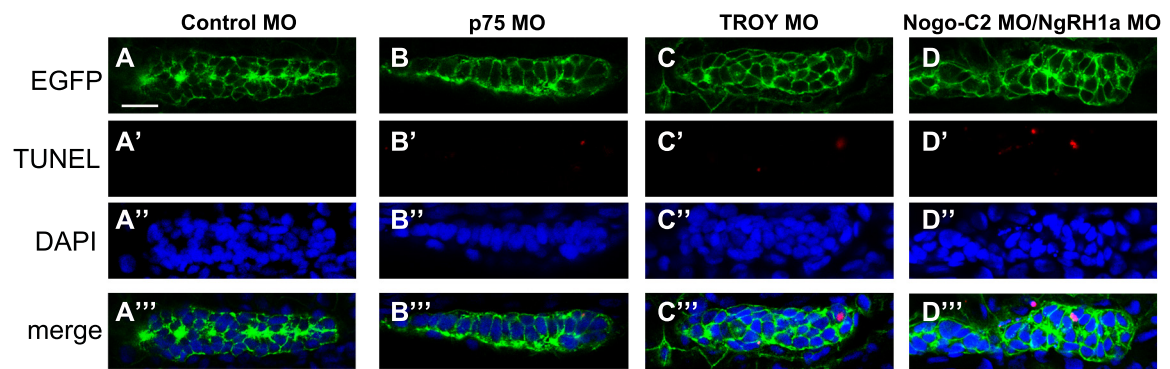

Figure S6

Supplement: Figure S6 — Non-specific MO-induced apoptosis was not observed in p75 , TROY and Nogo-C2/NgRH1a morphants. Each MO was injected into the CldnB::lynEGFP transgenic line, and then collected the morphants with malformed PLL primordium at 26–34 hpf for TUNEL assay. One embryo at 30 hpf, representative of a large sample (n = 10), was shown. Apoptotic cells in PLL primordium in control embryos (panels A–A′″), and p75 (B–B′″), TROY (C–C′″), and Nogo-C2/NgRH1a (D–D′″) morphants were revealed by TUNEL staining in red. Nuclei were also stained with DAPI. Scale bar, 20 µm. (PDF) [file pone.0086345.s006.pdf]

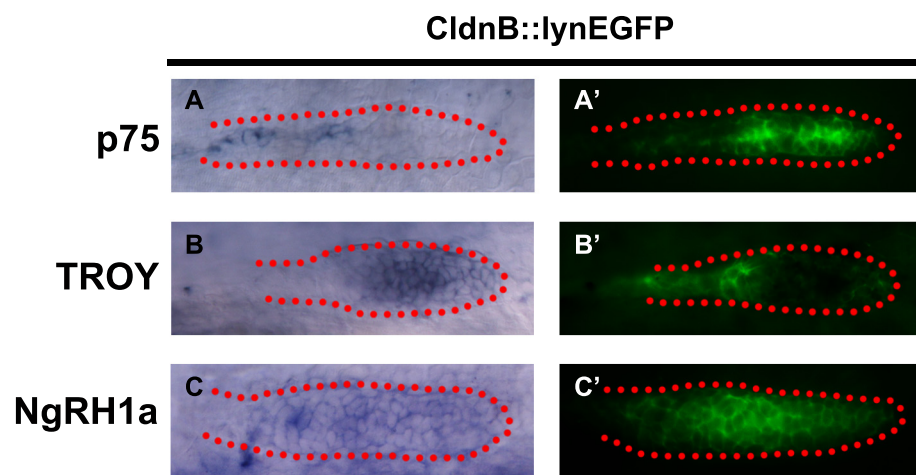

**Figure S7**

Supplement: Figure S7 — The mRNAs of p75, TROY and NgRH1a were expressed in the migrating primordium in zebrafish. Whole-mount in situ hybridization was performed with antisense probes against p75 (A), TROY (B), and NgRH1a (C) in CldnB::lynEGFP zebrafish at 30 hpf. The PLL primordium was simultaneously revealed by immunostaining with anti-GFP antibody (A′–C′). The PLL primordium is labeled with red dots. (PDF) [file pone.0086345.s007.pdf]
