# Supplementary material for: The Nogo-C2/Nogo Receptor Complex Regulates the Morphogenesis of Zebrafish Lateral Line Primordium through Modulating the Expression of dkk1b, a Wnt Signal Inhibitor
Source: PLoS One. 2014 Jan 21;9(1):e86345. doi: 10.1371/journal.pone.0086345 (PMC3897714; doi:10.1371/journal.pone.0086345)
Supplement: Figure S5 — Splice-blocking MO against Nogo-C2 and NgRH1a also disrupted PLL development in zebrafish. Zebrafish embryos were injected with splice-blocking MOs against either (A) Nogo-C2 or (B) NgRH1a, and the neuromasts were stained with 4-Di-2-ASP at 72 hpf (upper left panels). The numbers of PLL neuromasts in these morphants at 72 hpf are summarized (lower left panels). The MO dosages used and sample numbers (N) are indicated. The efficiency and specificity of Nogo-C2-SB-MO and NgRH1a-SB-MO were confirmed by RT-PCR with two primer sets, as illustrated in the top right panels. The sequences of splice-blocking MOs and primers used in RT-PCR are provided. (PDF) [file pone.0086345.s005.pdf]

A

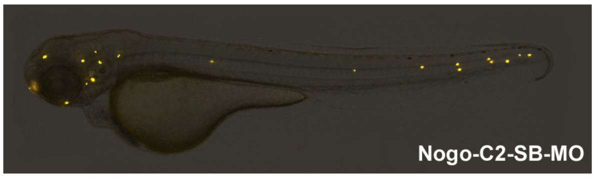

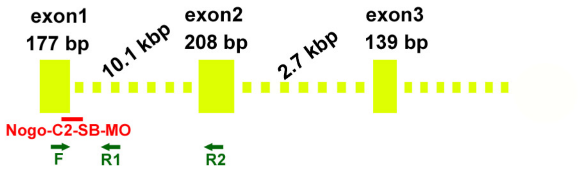

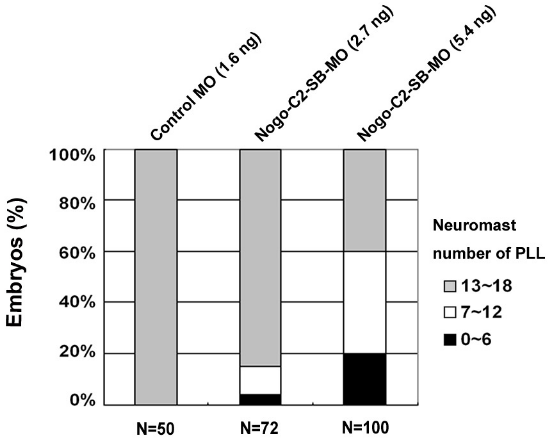

Sequences of Nogo-C2-SB-MO and primers used in RT-PCR

Nogo-C2-SB-MO	5'-ACGCCAAAACAAGCATATACCCTGT-3'
Primer-F	5'-GAGACTCGTGCCTCCTTCCTCAC-3'
Primer-R1	5'-CAACAGCATTCTCTGGCTGAGGG-3'
Primer-R2	5'-GCTAAGGGACAGCAGCAGGAAC-3'

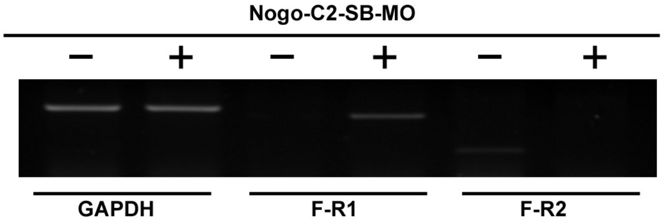

B

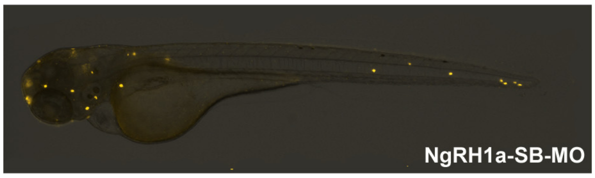

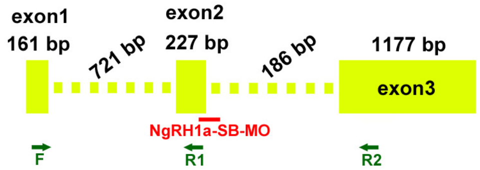

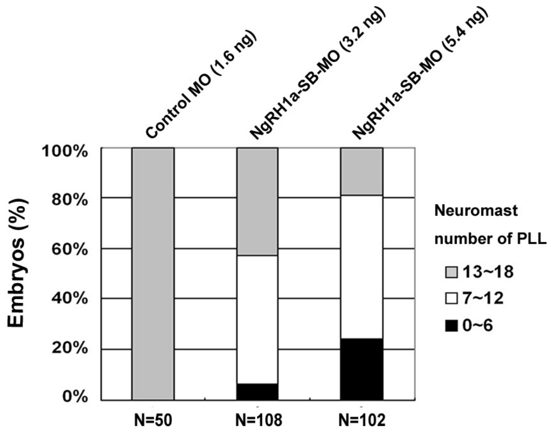

Sequences of NgRH1a-SB-MO and primers used in RT-PCR

NgRH1a-SB-MO	5'-ATATAAAGTTCCTTACCTGTGTGCC-3'
Primer-F	5'- GAGCATCAGCGGAGATAGCAGAG-3'
Primer-R1	5'-CCCACTCTCAGTTCAGTAATGCGG-3'
Primer-R2	5'-AGGCTCTGGAGCTTCTCTAGGCC -3'

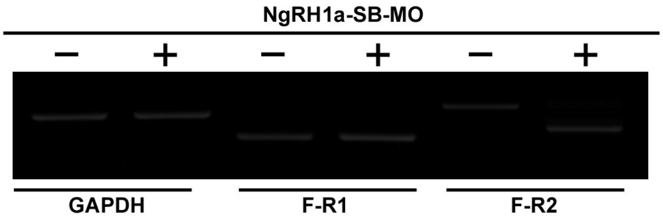

Figure S5
